# Identification and Comparative Analysis of Cadmium Tolerance-Associated miRNAs and Their Targets in Two Soybean Genotypes

**DOI:** 10.1371/journal.pone.0081471

**Published:** 2013-12-10

**Authors:** Xiaolong Fang, Yunyun Zhao, Qibin Ma, Yian Huang, Peng Wang, Jie Zhang, Hai Nian, Cunyi Yang

**Affiliations:** State Key Laboratory for Conservation and Utilization of Subtropical Agro-bioresources, College of Agriculture, South China Agricultural University, Guangzhou, China; National Taiwan University, Taiwan

## Abstract

MicroRNAs (miRNAs) play crucial roles in regulating the expression of various stress responses genes in plants. To investigate soybean (*Glycine max*) miRNAs involved in the response to cadmium (Cd), microarrays containing 953 unique miRNA probes were employed to identify differences in the expression patterns of the miRNAs between different genotypes, Huaxia3 (HX3, Cd-tolerant) and Zhonghuang24 (ZH24, Cd-sensitive). Twenty six Cd-responsive miRNAs were identified in total. Among them, nine were detected in both cultivars, while five were expressed only in HX3 and 12 were only in ZH24. The expression of 16 miRNAs was tested by qRT-PCR and most of the identified miRNAs were found to have similar expression patterns with microarray. Three hundred and seventy six target genes were identified for 204 miRNAs from a mixture degradome library, which was constructed from the root of HX3 and ZH24 with or without Cd treatment. Fifty five genes were identified to be cleaved by 14 Cd-responsive miRNAs. Gene ontology (GO) annotations showed that these target transcripts are implicated in a broad range of biological processes. In addition, the expression patterns of ten target genes were validated by qRT-PCR. The characterization of the miRNAs and the associated target genes in response to Cd exposure provides a framework for understanding the molecular mechanism of heavy metal tolerance in plants.

## Introduction

Heavy metal contamination in soil is a serious environment issue over the world, originating mainly from a set of anthropogenic activities, such as mining, industrial activities and utilization of phosphate fertilizers [1], [2]. In soil cadmium (Cd) can be easily taken up by plants, which results in various toxicity symptoms, such as reduced biomass, leaf chlorosis, inhibition of root growth, and morphological alterations, even plant death at excessive exposure [3]. In order to survive from stress environment, higher plants possess six possible ways to resist heavy metal exposure at the cellular level: (1) reduce metal bioavailability; (2) control metal influx; (3) chelate metals; (4) promote metal efflux; (5) compartmentalize and sequester metals; and (6) detoxify metal-induced reactive oxygen species (ROS) [4], [5]. Efforts have been made to identify genetic elements that are involved in Cd detoxification in plants. For example, the natural resistance-associated macrophage protein (NRAMP) transporter is reported to function in Cd uptake in *Arabidopsis thaliana* and rice (*Oryza sativa*) [6], [7]. On the contrary, heavy metal transporter 3 (HMA3) plays a role in sequestration of Cd into vacuoles in *Arabidopsis*, rice and soybean (*Glycine max*) [8]–[10]. In addition, two major quantitative trait loci for seed Cd accumulation, *Cda1* and *cd1*, have been well verified by Jegadeesan *et al.* [11] and Benitez *et al.* [10], [12], respectively. However, it is still unknown how the Cd tolerance is controlled by soybean genes or loci identified from these studies.

MiRNAs are one of the major types of endogenous, small, non-protein coding single-stranded RNA, around 22–24 nucleotides in higher plants, which regulate gene expression at the post-transcription and translation levels [13]–[15]. Numerous studies have demonstrated that miRNAs implicate in most of the essential physiological processes in plants, including development regulation, signal transduction, and stress responses [16], [17]. MiRNAs are important for plant responses to heavy metal stress [18]–[20]. For instance, miR393 and miR171 respond to heavy metals in *Brassica* (*B*. *napus*) [21], [22] and *Medicago* (*M*. *truncatula*) [23]. Additionally, 19 potential novel miRNAs responsive to Cd were isolated by using conventional sequencing approaches [24]. Recently, the genome-wide discovery of new miRNAs and analysis of the expression patterns becomes easier by using new small RNA sequencing and microarray technologies. Ding *et al.* [25] verified that miR166, miR171, miR390, miR156 and miR168 responded to Cd stress in rice, and Zhou *et al*. [26] reported that miR396, miR397, miR398 and miR408 were related to Cd exposure in *Brassica*.

To thoroughly characterize the function of each miRNAs, it is necessary not only to accurately identify the miRNAs, but also to validate the targets and to explore their interactions. Generally, according to the perfect sequence complementarity between a miRNA and its target(s) or the conservation of miRNA targets among different plant species, computational target prediction has been globally adopted to evaluate targets of miRNAs in plant [27], [28]. Nevertheless, due to the existence of the higher mismatch in miRNA-target pairing, computational target prediction is often questionable on the authenticity of predicted target transcripts [29]. Therefore, all the prediction targets need to be verified by experimental approaches. The modified 5′ RACE (rapid amplification of cDNA ends) has been widely applied in target confirmation and cleavage site mapping [30]. However, this method is used only in small-scale due to time and labor consuming and higher cost. Fortunately, degradome sequencing, a high-throughput approach known as PARE (parallel analysis of RNA ends), has been adopted to validate the miRNA spliced target transcripts [31]–[33]. Recently, this technology has been successfully used to identify miRNA targets in *Arabidopsis* [31], [32], rice [33], and soybean [34].

Soybean is one of the most important agriculture crops in the world. However, Cd pollution in soil affects the yield and quality of soybean seeds. Several soybean miRNAs have been found to play roles in response to biotic and abiotic stresses [35]–[37]. However, little is known about the Cd-responsive miRNAs in soybean, even less understanding on the targets of Cd-responsive miRNAs for the regulation. To understand the regulatory mechanism of miRNAs in response to Cd treatment, a custom-built miRNAs microarray chip was designed and used to detect the expression of miRNAs in HX3 and ZH24 roots under Cd stress or Cd-free. A total of 26 miRNAs were identified in response to Cd exposure in soybean roots. To evaluate the target transcripts of the miRNAs, a high-throughput degradome sequencing was adopted using a small RNA library consisting of four samples described above. Fifty five targets cleaved by 14 Cd-responsive miRNAs were identified. In addition, a number of Cd-responsive miRNAs and target mRNAs in soybean have been validated by quantitative RT-PCR (qRT-PCR).

## Materials and Methods

### Plant materials and stress treatment

Two contrasting soybean cultivars, HX3 (Cd-tolerant) and ZH24 (Cd-sensitive), were used in this research. Seeds of each cultivar were sown in sterile quartz sand and left for 4 days (d) to germinate. Then, seedlings were transferred into 1/2 full-strength nutrient solution containing 2.5 mM KNO_3_, 2.5 mM Ca(NO3)_2_^.^4H_2_O, 0.082 mM Fe(III)-EDTA, 4.57 μM MnCl_2_^.^4H_2_O, 0.25 mM K_2_SO_4_, 1.0 mM MgSO_4_^.^7H_2_O, 0.38 μM ZnSO_4_^.^7H_2_O, 1.57 mM CuSO_4_^.^5H_2_O, 0.09 μM (NH4)_6_MO_7_O_2_^.^4H_2_O, 23.13 μM H_3_BO_3_ and 0.5 mM KH_2_PO_4_, and then were cultured in a growth chamber with the following settings: 70% relative humidity, 28°C/25°C and a light regime of 14 h light/10 h dark. Two days after transplant, stress with different Cd concentrations (0 μM and 22 μM Cd added in culture solution) was carried out. The culture solutions, with or without Cd, were changed every two days. Roots were harvested at 6, 12, 24, 48, 96 and 144 h after the initiation of Cd treatment. The samples were flash-frozen in liquid nitrogen, and stored at −80°C.

### Microarray assay analysis

The miRNA microarray assays were performed by a service provider, LC Sciences in Houston, TX. The array design was based on 694 conserved miRNA mature sequences of legumes downloaded in miRBase Release 18.0 (http://www.mirbase.org/) and 259 well characterized miRNAs published in papers [38]–[43] (Table S1). Each probe was repeated three times on the chip to ensure reproducibility of microarray. The 5S rRNA was designed as the inner positive control; blank and non-homologous nucleic acids served as negative controls.

For each hybridization experiment, 2 to 5 μg total RNA sample, which was size fractionated using a YM-100 Microcon centrifugal filter (from Millipore) and the small RNAs (<300 nt) isolated were 3′-extended with a poly(A) tail using poly(A) polymerase. An oligonucleotide tag was then ligated to the poly(A) tail for later fluorescent dye staining; Hybridization was performed overnight on a μParaflo microfluidic chip using a micro-circulation pump (Atactic Technologies) [44]. On the microfluidic chip, each detection probe consisted of a chemically modified nucleotide coding segment complementary to target microRNA and a spacer segment of polyethylene glycol to extend the coding segment away from the substrate. The detection probes were made by *in situ* synthesis using PGR (photogenerated reagent) chemistry. The hybridization melting temperatures were balanced by chemical modifications of the detection probes. Hybridization used 100 μL 6xSSPE buffer (0.90 M NaCl, 60 mM Na_2_HPO_4_, 6 mM EDTA, pH 6.8) containing 25% formamide at 34°C. Hybridization images were collected using a laser scanner (GenePix 4000B, Molecular Device) and digitized using Array-Pro image analysis software (Media Cybernetics).

Data were analyzed by first subtracting the background and then normalizing the signals using a LOWESS filter (Locally-weighted Regression) [45]. To identify Cd-induced miRNAs, a criterion of signal >500, fold change >2 and *P*<0.01 was used.

### Microarray Data Deposition

All microarray data discussed in this publication have been deposited in NCBI's Gene Expression Omnibus and are accessible through GEO Series accession number GSE50388 (http://www.ncbi.nlm.nih.gov/geo/query/acc. cgi?acc  =  GSE50388).

### Validation of miRNAs expression profile via stem-loop qRT-PCR

The miRNA expression was examined by qRT-PCR experiments. Total RNA was isolated from roots of contrast and treated plants at 6, 12, 24, 48, 96 and 144h after Cd exposure and with TRIZOL reagent following the manufacturer's instructions (Invitrogen). RNA samples were treated with RNase-free DNase I (TaKaRa) to avoid amplification from genomic DNA. These samples were collected at the same time as those for miRNA microarray and degradome sequencing. The first cDNA strand was synthesized from total RNA using the MMLV-reverse transcriptase (Invitrogen) and miRNA specific stem loop primers, which were designed mostly according to the method described by Chen *et al.* [46] and Varkonyi-Gasic *et al.* [47]. Briefly, six to seven nucleotide tips pairing with the mature miRNA 3′ end were linked to a self-looped sequence (GTCGTATCCAGTGCGTGTCGTGGAGTCGGCAATTGCACTGGATACGAC) to make up the stem–loop reverse transcription primer. QRT-PCR analysis was carried out using the SsoFast EvaGreen Supermix kit (BIO-RAD) in a CXF96 (BIO-RAD) qRT-PCR System. Reaction conditions for thermal cycling were: 95°C for 3 min, 40 cycles of 95°C for 10 s, 57.0–63.3°C for 10 s and 72°C for 30 s. The annealing temperature (57.0–63.3°C) was adjusted to suit the amplification of the individual transcript. The sequences of stem–loop reverse transcriptase primers and miRNA-specific PCR primers are listed in Table S2. In each real qPCR experiment, each gene was run in triplicate with different cDNAs synthesized from three biological replicates. Relative fold changes of gene expression were calculated using the comparative ΔΔC_t_ method [48], and F-box was used as the reference gene [49]. Standard errors and standard deviations were calculated from replicates and significance was measured through Student's *t*-test at the level of 0.01<*P*≤0.05 and *P*≤0.01.

### Sequencing of degradome library and bioinformatics analysis

Construction of degradome library of soybean roots differed considerably from past efforts [31], [50] and followed as Ma et al. [51] with some modification. (1) Approximately 150 ng of poly(A)+ RNA was used as input RNA and annealing with Biotinylated Random Primers; (2) Strapavidin capture of RNA fragments through Biotinylated Random Primers; (3) 5′ adaptor ligation to only those RNAs containing 5′-monophosphates; (4) Reverse transcription and PCR; (5) Library was sequenced using the 5′ adapter only, resulting in the sequencing of the first 36 nucleotides of the inserts that represented the 5′ ends of the original RNAs. A Public software package, CleaveLand3.0 was used for analyzing sequencing data generated. All targets were classified into four categories based on the abundance of the resulting mRNA tag relative to the overall profile of degradome reads that matched the target [31], [50]. In category I, the abundance of cleavage sequences was equal to the most abundant degradome sequences on the transcript, and there was only one maximum on the transcript; in category II, the abundance of the degradome sequences at the cleavage site was equal to the maximum abundance on the transcript, and there was more than one maximum on the transcript; in category III, the abundance of cleavage sequences was less than the maximum but higher than the median for the transcript; in category IV, the abundance at cleavage site was equal to or less than the median for the transcript. The optimized score thresholds were set to 4.5 for category I, 4 for category II, 3.5 for category III, and 3 for category IV. These thresholds were used to select the resulting output. The gene ontology (GO) analysis of the candidate target transcripts of the identified miRNAs in this research was performed using the AgriGO toolkit [52].

### Degradome sequencing Data Deposition

All degradome sequencing data discussed in this publication have been deposited in NCBI's Gene Expression Omnibus and are accessible through GEO Series accession number GSE50063 (http://www.ncbi.nlm.nih.gov/geo/query/acc. cgi?acc  =  GSE50063).

### Validation of the miRNA target gene expression profile by qRT-PCR

The expression patterns of the target genes identified by degradome sequencing were evaluated by qRT-PCR. Total RNA was isolated from soybean root as the method described above. One µg of total RNA was used for initiating the reverse transcription reaction, and incubated with oligo(dT) primer. The reverse transcription reaction was performed by MMLV-reverse transcriptase (Invitrogen) following the supplier's manual. Then, target gene primers were added to carry out the PCR array. A soybean housekeeping gene F-box [49] was used for the reference gene for qRT-PCR. Supplementary Table S3 shows the primer sequencing of the target gene and the F-box gene. In each qPCR experiment, each gene was run in triplicate with different cDNAs synthesized from three biological replicates. The ΔΔC_t_ method was used to determine the expression level differences among samples of root with Cd treatment and without Cd treatment [48]. Standard errors and standard deviations were calculated from replicates and significance was measured through Student's *t*-test at the level of 0.01<*P*≤0.05 and *P*≤0.01.

## Results

### Identification and expression patterns of Cd-responsive miRNAs in soybean

In this study, miRNA microarrays containing 953 miRNA mature sequences were used to investigate the expression patterns of miRNAs in soybean roots under Cd stress (microarray data were deposited into the NCBI-GEO with accession no. GSE50388). There were 34 miRNAs in HX3 and 48 miRNAs in ZH24 which differentially expressed between Cd treatment and control with at least one signal >500 and *P*<0.01. To identify Cd-responsive miRNAs, a further strict criterion with fold change >2 was used. A total of 26 miRNAs were identified as Cd-responsive miRNAs (Table 1). Among those miRNAs, 16 miRNAs from *G. max*, two miRNAs from wild soybean (*Glycine soja*), two miRNAs from *Arachis hypogaea*, and one miRNA from *Vigna unguiculata* were released in miRBase, while one miRNA (Gma-m040-5p) from *G. max* [42], three miRNAs (PN-miR397a_L-1, PN-miR1509b_R+1 and PC-15-5p) from *G. soja* [43] and one miRNA (Vun78330_1521_100) from *V. unguiculata* [41] were newly identified as novel miRNA in each species, respectively. According to the similarity of the sequences, Gma-m040-5p was characterized as one member of miRNA390 family, Vun78330_1521_100 was identified as one member of miRNA319 family, while, PC-15-5p, having 25 nucleotides, shared 14 common sequences with gma-miR6300 from 3 to 18.

**Table 1:** Cd-responsive miRNAs identified by microarray analysis.

	Huaxia3				Zhonghuang24				
miRNA	CK	Cd	fold change log2(Cd/CK)	p-value	CK	Cd	fold change log2(Cd/CK)	p-value	mature Sequence (5′ to 3′)
gma-miR3522	625	4,597	2.88	1.94E-04	451	2,981	2.72	4.06E-05	AGACCAAAUGAGCAGCUGA
gso-miR3522a	647	4,257	2.72	7.37E-04	437	3,087	2.82	2.13E-05	UGAGACCAAAUGAGCAGCUGA
gso-miR3522b	661	4,277	2.69	4.48E-05	465	3,127	2.75	2.39E-05	UGAGACCAAAUGAGCAGCUGAC
gma-miR397a	237	1,535	2.70	3.06E-05	152	866	2.51	4.69E-03	UCAUUGAGUGCAGCGUUGAUG
PN-miR397a_L-1	212	1,408	2.73	1.79E-03	169	847	2.33	1.53E-02	CAUUGAGUGCAGCGUUGAUG
ahy-miR408-3p	446	2,955	2.73	5.64E-05	422	1,835	2.12	6.04E-04	AUGCACUGCCUCUUCCCUGGC
gma-miR408	752	5,178	2.78	2.78E-05	745	3,390	2.19	2.58E-03	UGCACUGCCUCUUCCCUGGC
gma-miR408b-5p	983	3,978	2.02	2.57E-05	603	2,414	2.00	1.86E-05	CUGGGAACAGGCAGGGCACG
gma-miR4996	2,412	9,556	1.99	8.01E-04	4,720	9,638	1.03	1.70E-04	UAGAAGCUCCCCAUGUUCUC
gma-miR396a-3p	1,839	2,175	0.24	1.23E-02	1,084	2,414	1.16	4.43E-04	UUCAAUAAAGCUGUGGGAAG
gma-miR396i-3p	2,038	2,461	0.27	4.25E-02	1,253	2,706	1.11	3.78E-03	GUUCAAUAAAGCUGUGGGAAG
ahy-miR398	435	1,151	1.40	1.97E-03	462	730	0.66	6.79E-04	UGUGUUCUCAGGUCACCCCU
gma-miR398a	399	868	1.12	4.86E-04	440	578	0.39	1.43E-01	UGUGUUCUCAGGUCACCCCUU
gma-miR398c	270	1,171	2.12	3.19E-03	374	638	0.77	4.08E-03	UGUGUUCUCAGGUCGCCCCUG
gma-miR1535b	150	305	1.02	4.22E-03	658	323	−1.03	1.34E-03	CUUGUUUGUGGUGAUGUCUAG
Gma-m040-5p	515	544	0.08	3.49E-01	726	350	−1.05	3.90E-03	AAGCUCAGGAGGGAUAGCACCA
gma-miR390a-5p	575	625	0.12	3.56E-02	834	349	−1.26	4.80E-04	AAGCUCAGGAGGGAUAGCGCC
gma-miR4403	330	693	1.07	3.68E-04	726	451	−0.69	2.31E-03	ACGGACACCGAACACGACACGGAC
gma-miR1509b	4,704	2,780	−0.76	5.62E-03	6,728	2,970	−1.18	5.49E-04	UUAAUCAAGGAAAUCACGGUU
PN-miR1509b_R+1	5,078	3,474	−0.55	1.44E-02	7,284	3,595	−1.02	5.09E-03	UUAAUCAAGGAAAUCACGGUUG
gma-miR5037b	1,751	1,397	−0.33	5.84E-02	2,623	1,269	−1.05	3.41E-04	AACCCUCAAAGGCUUCCUAG
PC-15-5p	1,520	1,374	−0.15	2.18E-01	2,416	1,145	−1.08	4.42E-04	UCCGUUGUAGUCUAGUUGGUUAGGA
gma-miR396b-5p	339	228	−0.57	5.40E-03	1,471	394	−1.90	7.67E-04	UUCCACAGCUUUCUUGAACUU
vun-miR319b	1,366	523	−1.38	5.18E-03	1,878	737	−1.35	1.98E-04	CUUGGACUGAAGGGAGCUCCU
gma-miR319c	346	176	−0.98	1.17E-03	518	182	−1.51	2.19E-04	UUGGACUGAAAGGAGCUCCU
Vun78330_1521_100	366	96	−1.93	6.32E-04	626	180	−1.80	5.00E-05	UUUGGACUGAAGGGAGCUCCU

*P*<0.01 were treated as Cd-responsive miRNAs. *a*, PN means “potential novel'; *b*, PC means “Predicted Candidate”; *c*. gma/Gma mean “*Glycine max*”; *d.* gso means “*Glycine soja*”; *e*. ahy means “*Acacia mangium*”; *f*. vun means “*Vigna unguiculata*”. MiRNAs with at least one signal >500 and fold change >2 between Cd treatment and control and

Out of 26 miRNAs, nine were identified in the both cultivars, five were identified only in HX3 and 12 miRNAs were found only in ZH24 (Table 1). There were 13 up-regulated miRNAs and only one down-regulated miRNA (vun-miR319b) in HX3. However, the number of down-regulated miRNAs in ZH24 (with 11 down-regulated miRNAs and ten up-regulated miRNAs) was much more than that in HX3. Among the 26 miRNAs, 14 miRNAs (gma-miR3522, gso-miR3522a, gso-miR3522b, gma-miR397a, PN-miR397a_L-1, ahy-miR408-3p, gma-miR408, gma-miR408b-5p, gma-miR4996, gma-miR396a-3p, gma-miR396i-3p, ahy-miR398, gma-miR398a and gma-miR398c) belonging to six families were up-regulated by Cd exposure (*P*<0.01) in both HX3 and ZH24. Ahy-miR398, gma-miR398a and gma-miR398c, of 398 family members, showed over 2-fold up-regulated only in HX3, whereas two 396 family members, miR396a-3p and miR396i-3p showed over 2-fold up-regulated only in ZH24. Meanwhile, eight miRNAs (gma-miR1509b, PN-miR1509b_R+1, gma-miR5037b, PC-15-5p, gma-miR396b-5p, vun-miR319b, gma-miR319c and Vun78330_1521_100) belonging to five families were identified to be down-regulated in both genotypes, but only in ZH24 with over 2-fold change response to Cd stress. In addition, four miRNAs (Gma–m040-5p, gma-miR1535b gma-miR4403 and miR390a-5p) showed different expression patterns in two genotypes, in which, gma-miR4403 were over 2-fold change up-regulated only in HX3, but others were down-regulated with over 2-fold change only in ZH24. It was worthy notice that three members of miR396 family performed different expression, in which miR396a-3p and miR396i-3p were significantly up-regulated, while miR396b-5p was down-regulated (Table 1).

There are some miRNAs which were not characterized as Cd-responsive miRNAs should be noticed. Fourteen miRNAs (PC-34-5p, gso-mir1510b-p5, ahy-miR156a, ahy-miR156c, PN-mir156f-p3, PN-miR160c_1ss15AC, PN-miR164a_L-1, PN-miR166_L-1, ahy-miR167-5p, gso-miR2109, aau-miR319, aau-miR396, PN-miR5072_L-4, and PC-33-3p) of 12 miRNA families from other species had strong signals (signal >1000) in microarray (Table S4), in which eight miRNAs (gso-mir1510b-p5, ahy-miR156a, ahy-miR156c, PN-miR164a_L-1, PN-miR166_L-1, gso-miR2109, aau-miR319, and aau-miR396) had similar sequences with soybean miRNA in mirBase only added, lost, or changed one base at 3′ or 5′ end, whereas six miRNAs (PC-34-5p, PN-mir156f-p3, PN-miR160c_1ss15AC, ahy-miR167-5p, PN-miR5072_L-4, and PC-33-3p) had no similar soybean sequences in miBase. These results suggest that some new miRNAs may play important roles in responding to Cd in these two genotypes.

### Confirmation of differentially expressed miRNAs by qRT-PCR

**Figure 1:**
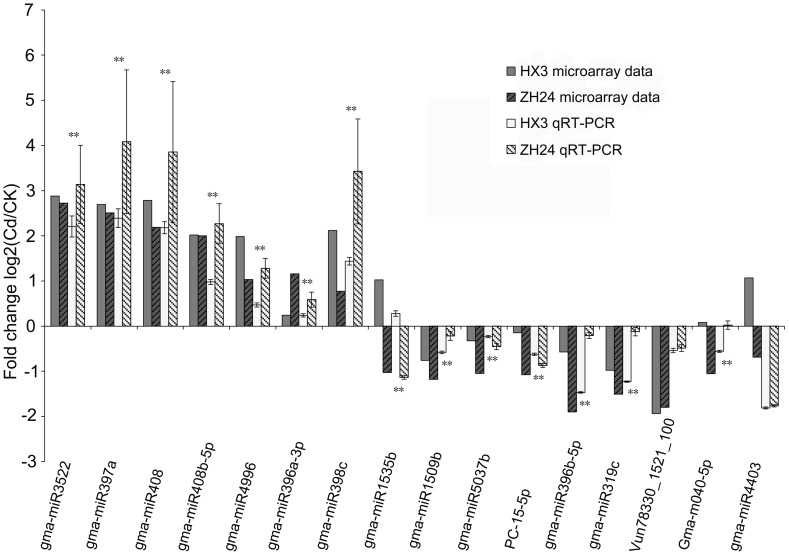
Expression patterns of 16 Cd-responsive miRNAs confirmed by qRT-PCR. The *y*-axis represents the expression levels of Cd/CK. The dark grey shading bar represents the relative expression level of miRNAs in HX3 measured by microarray in responsive to Cd stress. The dark grey diagonal stripe shading bar represents the relative expression level of miRNAs in ZH24 measured by microarray in responsive to Cd stress. The french grey shading bar represents the relative expression level of miRNAs in HX3 measured by qRT-PCR in responsive to Cd stress. The french grey diagonal stripe shading bar represents the relative expression level of miRNAs in ZH24 measured by qRT-PCR in responsive to Cd stress. The results of qRT-PCR are mean ± SD of duplicates of three biological replicates. Significance of the changes between HX3 and ZH24 at the levels of Cd/CK was checked with Student's t-test at the level of P≤0.01 (shown as “**”).

To validate the expression profile of the soybean miRNAs obtained through the microarray assays, qRT-PCR was performed for 16 Cd-responsive miRNAs (gma-miR3522, gma-miR397a, gma-miR408, gma-miR408b-5p, gma-miR4996, gma-miR396a-3p, gma-miR398c, gma-miR1509b, gma-miR5037b, PC-15-5p, gma-miR319c, gma-miR1535b, Gma-m040-5p, gma-miR4403, gma-miR396b-5p, and Vun78330_1521_100) in the same samples used in the microarray (Figure S1 and S2). The results from qRT-PCR were compared with those from the microarray (Figure 1). The expression patterns of all 16 Cd-responsive miRNAs obtained by qRT-PCR were mostly similar to the results from the microarray, except for those of Gma-m040-5p and gma-miR4403. The expression patterns of Gma-m040-5p and gma-miR4403 obtained by qRT-PCR in HX3 were not consistent with that fromthe microarray but were similar with that in ZH24. Moreover, gma-miR1535b was confirmed to be up-regulated in HX3 and down-regulated in ZH24, and miR396b-5p was verified to be down-regulated in both cultivars and showed different trends compared with miR396a-3p. Although the fold change of expression valued by qRT-PCR was not exactly identical to that calculated by the microarray, the differential expression trends were the same by both methods. The qRT-PCR results ultimately reflected consistency with the microarray data.

### Identification of targets of Cd-responsive miRNAs in soybean

MiRNA target confirmation is a prerequisite to get deep understanding of the functional role of miRNAs. Only several targets of miRNAs from soybean have been predicted [35]–[38], [53]. To identify more targets in soybean, particularly specific targets of Cd-stress-responsive miRNAs, high-throughput degradome sequencing was used (the degradome sequencing data were deposited into the NCBI-GEO with accession no. GSE50063). In total, we obtained 8913111 raw reads from the library which was constructed from a mixture of four samples (HX3-CK, HX3-Cd-treatment, ZH24-CK and ZH24-Cd-treatment). After removing the reads without the CAGAG adaptor, 5430126 unique raw-reads were obtained. The unique sequences were aligned to the *G. max* genome database, and 6516276 reads were mapped to the genome. The mapped reads from the library represented 51481 annotated *G. max* genes. The sliced target transcripts were categorized into four groups according to the relative abundance of the tags at the target mRNA sites.

**Figure 2:**
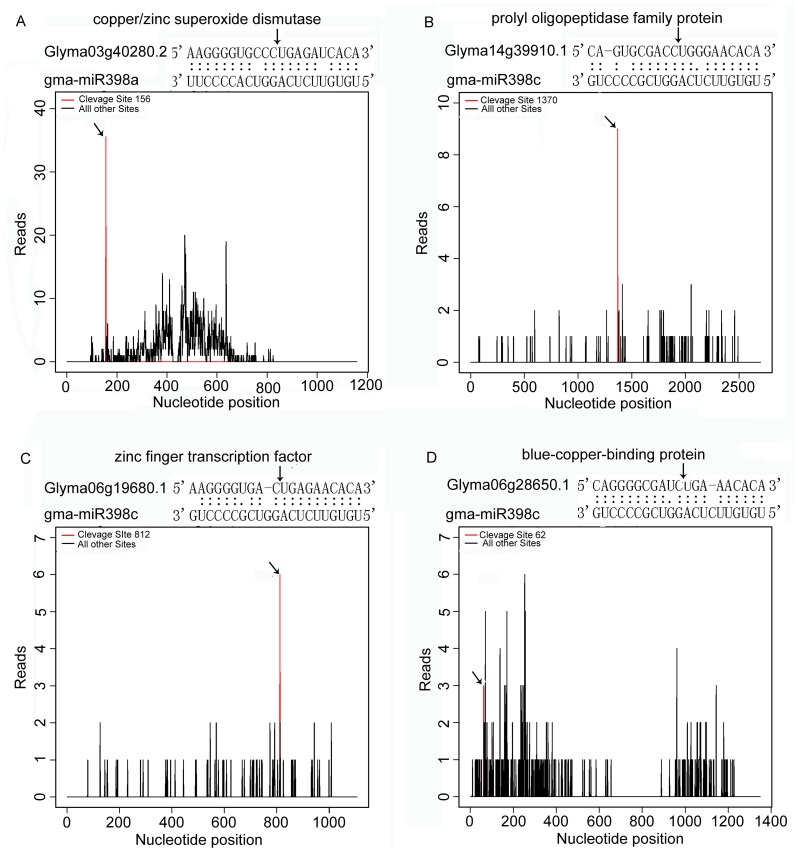
T-plots of the targets cleaved by the miR398 family. The T-plots show the distribution of the degradome tags along the full-length of the target mRNA sequence (bottom). The red line represents the sliced target trancripts and is shown in arrow. The alignment shows the miRNA with a portion of its target sequence (top). The two dots indicate matched RNA base pairs; one dot indicates a GU mismatch. The arrow shows the cleavage site. (A) Examples of copper/zinc superoxide dismutase as target for gma-miR398a. (B) Examples of prolyl oligopeptide family protein as target for gma-miR398c. (C) Examples of zinc finger transcription factor as target for gma-miR398c. (D) Examples of blue-copper-binding protein as target for gma-miR398c.

In total, 376 targets that could potentially be cleaved by 204 miRNAs were identified (Table S5). There were 193, 22, 210 and 4 targets in categories I, II, III and IV respectively. Most of miRNAs cleaved two or more different transcription targets, while 35 miRNAs (17.16%) were detected to cleave only one transcription target. Twenty five transcriptions were identified to be cleaved by mtr-miR169m, which was the highest amount of transcriptions cleaved by the same miRNA in this study. Furthermore, gma-miR396e was identified to cleave 19 members of growth-regulating factor families and a gene (Glyma03g36370.1) encoding a protein of unknown function. Most targets for conserved miRNAs were conserved, but still some conserved miRNAs had non-conserved or novel transcripts. For instance, a transcript encoding GATA type zinc finger transcription factor family protein was identified for gma-miR398 (T-plots of the four of the targets are presented in Figure 2).

**Figure 3:**
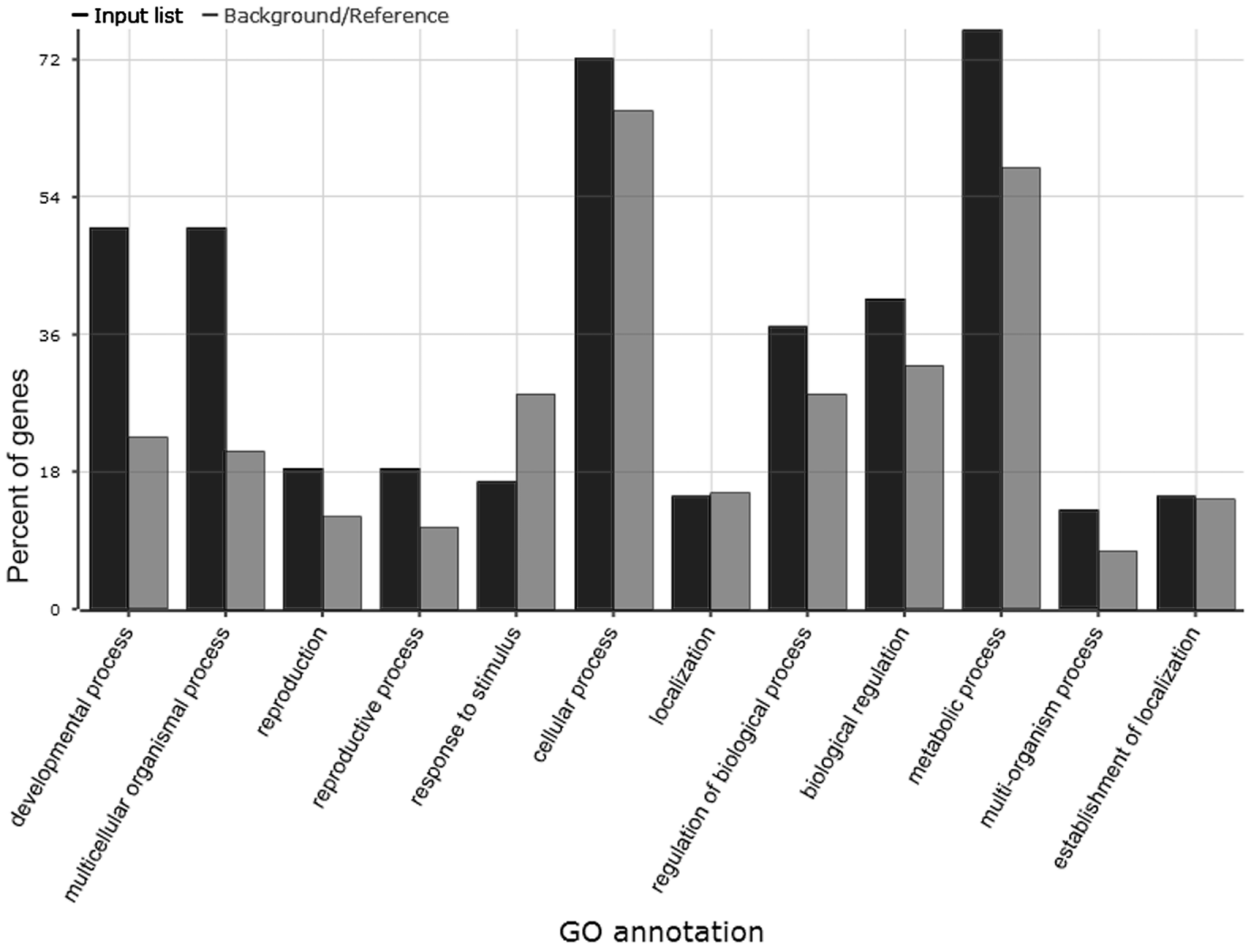
GO analyses of the targets of the 14 Cd-stress-responsive miRNAs in soybean. The dark grey shading bars indicate the enrichment of the GO terms in the miRNA targets in GO. The french grey shading bars indicate the percentage of the total annotated soybean genes that were mapped to the GO terms.

Fourteen out of the 26 Cd-stress-responsive miRNAs (9 miRNA families) were identified to cleave 55 transcripts by the degradome sequencing (Table 2). Gene ontology (GO) analysis was carried out by using the AgriGO toolkit [52]. The results revealed that 54 genes were annotated as being involved in 12 biological processes (Figure 3). More than 72% of targets took part in cellular and metabolic processes. It is notable that 20 targets (37.04%) were involved in regulation of biological process which was more enriched in the targets of Cd-responsive miRNA than in the soybean genes as a whole. In contrast, the proportion of the Cd-responsive miRNA targets in response to stimulus (16.67%) was less than that of the whole soybean genes.

**Table 2:** Target genes of 14 Cd-responsive miRNAs and their functional annotation.

miRNA Family	Target	AlignmentScore	AlignmentRange	CleavageSite	Category	Annotation
miR397	Glyma06g43700.1	0.5	587–607	598	0	Laccase/Diphenol oxidase family protein
	Glyma18g07240.1	0.5	636–656	647	0	Laccase/Diphenol oxidase family protein
	Glyma18g42520.1	0.5	669–689	680	0	Laccase/Diphenol oxidase family protein
	Glyma03g15800.1	1	720–740	731	0	laccase 7
	Glyma03g15800.2	1	669–689	680	0	laccase 7
	Glyma12g14230.1	0.5	669–689	680	2	Laccase/Diphenol oxidase family protein
	Glyma03g14450.1	1	579–599	590	2	Laccase/Diphenol oxidase family protein
	Glyma03g15800.3	1	720–740	731	2	laccase 7
	Glyma01g27710.1	1.5	754–774	765	2	Laccase/Diphenol oxidase family protein
miR408	Glyma03g26060.2	1.5	440–459	450	0	Cupredoxin superfamily protein
	Glyma04g42120.1	1.5	6–26	17	0	plantacyanin
	Glyma06g12680.1	1.5	5–25	16	0	plantacyanin
	Glyma05g30380.1	2.5	34–53	44	0	plantacyanin
	Glyma08g13510.1	2.5	201–220	211	0	plantacyanin
	Glyma14g06070.1	4	51–71	62	1	laccase 5
	Glyma03g26060.1	1.5	682–701	692	2	uclacyanin 1
miR4996	Glyma16g02340.1	3.5	922–941	932	2	CDPK-related kinase 3
	Glyma07g05750.1	3.5	882–901	892	2	CDPK-related kinase 3
miR398	Glyma06g19680.1	2	802–821	812	0	GATA type zinc finger transcription factor family protein
	Glyma14g39910.1	3	1360–1379	1370	0	Prolyl oligopeptidase family protein
	Glyma14g39910.2	3	1360–1379	1370	0	Prolyl oligopeptidase family protein
	Glyma14g39910.3	3	1360–1379	1370	0	Prolyl oligopeptidase family protein
	Glyma03g40280.2	4	146–165	156	0	copper/zinc superoxide dismutase 1
	Glyma03g40280.3	4	146–165	156	0	copper/zinc superoxide dismutase 1
	Glyma06g28650.1	3	51–70	62	2	blue-copper-binding protein
miR1535	Glyma17g02080.1	2	166–186	177	2	isopentenyltransferase 5
	Glyma07g38620.1	2	168–188	179	2	isopentenyltransferase 5
miR1509	Glyma18g03980.2	2	2144–2164	2155	0	Protein of unknown function (DUF1644)
miR5037	Glyma18g04770.1	2.5	1066–1085	1076	2	ARM repeat superfamily protein
	Glyma11g33450.1	3.5	1149–1168	1159	2	CYS, MET, PRO, and GLY protein 1
miR396	Glyma01g34650.1	4	117–138	128	0	growth-regulating factor 9
	Glyma01g44470.1	4	417–438	428	0	growth-regulating factor 5
	Glyma03g02500.1	4	539–560	550	0	growth-regulating factor 9
	Glyma09g07990.1	4	369–390	380	0	growth-regulating factor 5
	Glyma10g07790.1	4	543–564	554	0	growth-regulating factor 4
	Glyma11g01060.1	4	338–359	349	0	growth-regulating factor 5
	Glyma11g11820.1	4	375–396	386	0	growth-regulating factor 5
	Glyma12g01730.1	4	493–514	504	0	growth-regulating factor 5
	Glyma12g01730.2	4	493–514	504	0	growth-regulating factor 5
	Glyma14g10090.1	4	693–714	704	0	growth-regulating factor 1
	Glyma14g10100.1	4	700–721	711	0	growth-regulating factor 1
	Glyma15g19460.1	4	336–357	347	0	growth-regulating factor 5
	Glyma16g00970.1	4	342–363	353	0	growth-regulating factor 5
	Glyma17g35090.1	4	902–923	913	0	growth-regulating factor 1
	Glyma17g35100.1	4	713–734	724	0	growth-regulating factor 1
	Glyma19g37740.2	4	315–336	326	0	growth-regulating factor 4
	Glyma19g37740.1	4	860–881	871	1	growth-regulating factor 4
	Glyma03g31420.1	3.5	2026–2046	2037	2	ATPase E1-E2 type family protein/haloacid dehalogenase-like hydrolase family protein
miR319	Glyma03g38120.1	4	2073–2093	2084	0	HCO3- transporter family
	Glyma13g04030.1	4	752–772	763	0	myb domain protein 33
	Glyma13g25720.1	4	827–847	838	0	myb domain protein 33
	Glyma15g35860.1	4	926–946	937	0	myb domain protein 33
	Glyma19g40720.1	4	2151–2171	2162	0	HCO3- transporter family
	Glyma20g11040.1	4	907–927	918	0	myb domain protein 33
	Glyma05g27370.1	3.5	912–932	923	1	TEOSINTE BRANCHED 1, cycloidea and PCF transcription factor 2

The numbers represent the four categories as follows: 0 indicates category I; 1 indicates category II; 2 indicates category III; 3 indicates category IV.

Seven out of 14 miRNAs which were all up-regulated in HX3 and ZH24 were identified to cleave 25 targets belonging to nine families as blue-copper-binding protein family, CDPK-related kinase family, copper/zinc superoxide dismutase family, cupredoxin superoxide dismutase family, GATA type zinc finger transcription factor family, laccase (LAC) family, plantacyanin family, prolyl oligopetidase family and uclacyanin family. Six down-regulated miRNAs in HX3 and ZH24 were found to slice 27 transcriptions belonging to ARM repeat superfamily, ATPase E1-E2 type family, CYS, MET, PRO, and GLY protein family, growth-regulating factor family, myb domain protein family, HCO3- transporter family and cycloidea and PCF transcription factor family and to cleave one target (Glyma18g03980.2) with unknown function. Two members of isopentenyltransferase gene family were found to be cleaved by gma-miR1535b which was up-regulated in HX3 but down-regulated in ZH24.

### Confirmation of target transcripts of Cd-responsive miRNAs

**Figure 4:**
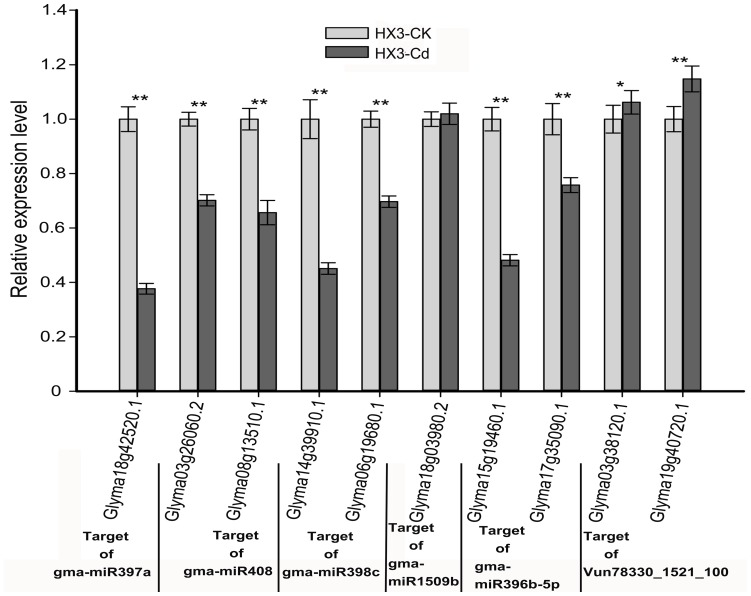
Expression profiles of target genes of Cd-responsive miRNAs between HX3-CK and HX3-Cd. The expression levels of mRNAs were normalized to the level of F-box. The results are mean ± SD of the duplicates of three biological replicates. Significance of the changes between HX3-CK and HX3-Cd was checked with Student's *t*-test at the level of 0.01<*P*≤0.05 (shown as “*”) and *P*≤0.01 (shown as “**”).

**Figure 5:**
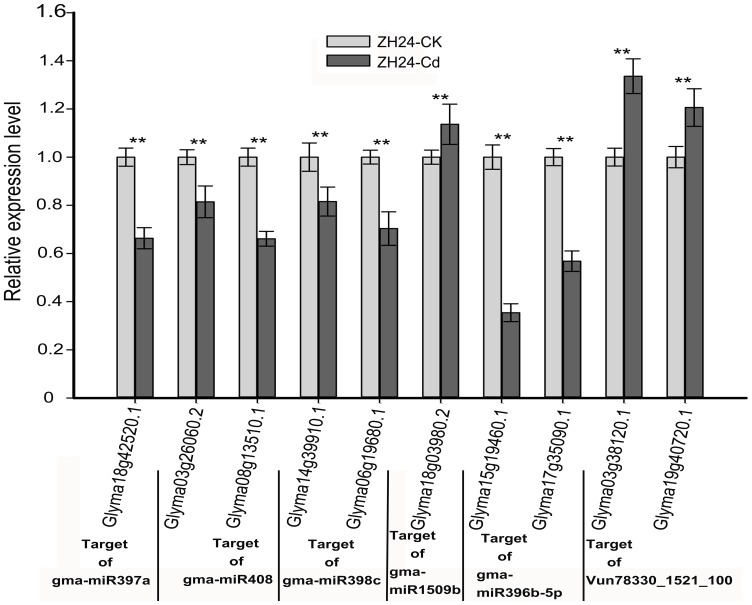
Expression profiles of target genes of Cd-responsive miRNAs between ZH24-CK and ZH24-Cd. The expression levels of mRNAs were normalized to the level of F-box. The results are mean ± SD of the duplicates of three biological replicates. Significance of the changes between ZH24-CK and ZH24-Cd was checked with Student's *t*-test at the level of 0.01<*P*≤0.05 (shown as “*”) and *P*≤0.01 (shown as “**”).

To investigate whether the target genes detected by the degradome sequencing were actually regulated by Cd-responsive miRNAs, the expression levels of ten targets (Glyma18g42520.1, Glyma03g26060.2, Glyma08g13510.1, Glyma14g39910.1, Glyma06g19680.1, Glyma18g03980.2, Glyma15g19460.1, Glyma17g35090.1, Glyma03g38120.1 and Glyma19g40720.1) of six Cd-responsive miRNAs (gma-miR397a, gma-miR408, gma-miR398c, gma-miR1509b, gma-miR396b-5p and Vun78330_1521_100) were measured in Cd tolerant (HX3) and Cd sensitive (ZH24) soybean roots exposed to 22 μM Cd with a mixture of samples from 6 h to 144 h by using qRT-PCR (Figures 4 and 5). As shown in Figures 1, S1 and S2, miRNA397a, miRNA408 and miRNA398c all showed significantly up-regulated in both HX3 and ZH24, meanwhile their corresponding targets, Glyma18g42520.1, Glyma03g26060.2, Glyma08g13510.1 Glyma14g39910.1 and Glyma06g19680.2 (Figures 4 and 5), showed apparently down-regulated alteration in the two cultivars. For miR1509b and Vun78330_1521_100, both of which showed apparently down-regulation in HX3 and ZH24 with Cd treatment (Figures S1and S2), their targets, Glyma18g03980.2, Glyma03g38120.1 and Glyma19g40720.1, showed a little higher up-regulation in ZH24 than that in HX3 (Figures 4 and 5). While the expression of gma-miR396b-5p showed indirectly correlation with their target genes (Figures 4 and 5). As shown in Figures 1, S1 and S2, miRNA396b displayed significantly down-regulated in HX3 and ZH24, whereas Glyma15g19460.1, Glyma17g35090.1, two targets of miRNA396b identified by degradome sequence, showed both down-regulated in the two cultivars (Figures 4 and 5).

## Discussion

Heavy metal, especially Cd, is toxic to plants and plants initiate a variety of tolerant strategies to overcome the Cd toxicity. Recently, increasing evidences have revealed that miRNAs played the crucial role on regulation of plant genes at the post-transcriptional in responding to metals stresses. Under Cd stress, miR397, miR408 and miR398 were reported to be up-regulated and miRNA396 and miRNA319 to be down-regulated in response to Cd exposure in the root of rice or *Brassica* [25], [26]. In this study, a total of 26 Cd-responsive miRNAs were identified; almost half of which, such as gma-miR397a, PN-miR397a_L-1, ahy-miR408-3p, gma-miR408b-5p, gma-miR408b-5p, gma-miR396b-5p, ahy-miR398, gma-miR398a, gma-miR398c, vun-miR319b, gma-miR319c and Vun78330_1521_100, have the similar alteration patterns in HX3 and ZH24 as that of rice and *Brassica* mentioned above (Table 1 and Figure 1). In addition, we also found that two other miR396 family members, gma-miR396a-3p and gma-miR396i-3p, showed up-regulated in the both cultivars, which was contrary with that of gma-miR396b-5p and its homologous members. The expression patterns of gma-miR396b-5p in two soybean genotypes were very similar with that of miR396 in rice [25] and *Brassica* [26]. For gma-miR390a-5p, an apparently down-regulated miRNA in ZH24, it has the same alteration with that in the root of rice [25] and shoot of *Brassica* [26], but was contrary with that in root tissue of *Brassica* [26]. It has been found so far that some miRNAs, including gma-miR3522, gso-miR3522a, gso-miR3522b, gma-miR4996, gma-miR1535b, Gma-m040-5p, gma-miR4403, gma-miR1509b, PN-miR1509b_R+1, gma-miR5037b and PC-15-5p, were responsive to Cd only in soybean, in which, gso-miR3522a, gso-miR3522b (released in miRBase 18.0), PN-miR1509b_R+1 and PC-15-5p [43], were found before only in wild soybean.

Of the 26 miRNAs identified in our study, almost all of them showed similar expression patterns in HX3 and ZH24, except for gma-miR1535b, which was detected as being up-regulated in HX3 and down-regulated in ZH24. The similarly of miRNA regulation may represent the fundamental mechanism of adapting to Cd exposure, and the difference of regulated miRNAs, including those which had distinct expression trends or had the same alteration but showed apparent expression level, may explain the distinct Cd sensitivities between the two soybean cultivars.

Usually, miRNAs regulate the expression of target genes at the post-transcriptional level by mediating gene silencing in plants. Large amount of target genes were also predicted by computer arithmetic, while quite a few of which have been validated by experiment, like 5′-RACE assay [53]. In this study, degredome library was prepared from root samples of two genotypes with or without Cd stress. Sequencing and analysis resulted in the identification of 376 targets for 204 miRNAs. Among these, 55 genes cleaved by 14 Cd-stress-responsive miRNAs were verified by degradome sequencing (Table 2). qRT-PCR results revealed that the expression patterns of five miRNAs were significantly negatively correlated with their corresponding target genes. (Figures 4 and 5).These results suggest that miRNAs play crucial roles in response to Cd by mediating target genes silencing.

Many abiotic conditions including heavy metal result in oxidative stress in plants [54]. Several miRNAs are involved in the regulation of genes responsible for antioxidation. MiR398 is the first miRNA identified to regulate plant responses to oxidative stress [55]. The *MIR398* promoter contains the GTAC motif that has an important feature in Cu responsiveness in *Arabidopsis* [56]. This motif is recognized by the SPL7 (SQUAMOSA promoter binding protein-like 7) transcription factor that directly binds to the promoter and activate transcription of *MIR398* gene. SPL7 is homologous to copper response regulator1, the transcription factor required for Cu deficiency response in *Chlamydomonas reinhardtii* [57]. In addition, SPL7 is required for the expression regulation of other Cu-related miRNAs such as miR397, miR408, and miR857 [56]. In this study, miR397a, miR408 and miRNA398c showed almost the similar up-regulated alteration to response to Cd exposure, which might imply that SPL7 involved in regulation of Cu deficiency and Cd response in soybean (Figures 1, S1 and S2).

Three most abundant copper proteins, copper/zinc superoxide dismutase (Cu, Zn-SOD; CSD), plastocyanin (PC) and cytochrome c oxidase (COX) are essential for oxidative stress alleviation, photosynthesis and respiratory electron transport, respectively [58]. CSD which is one target gene of miR398, as ROS scavenger and a major copper protein, is important for stress tolerance and survival in plant [59]. The mode of Cu-regulating ROS signal transduction was well studied in *A. thaliana* [60], [61]. Under high Cu stress, oxidative stress suppresses miR398 expression, which is essential for the accumulation of *CSD1* and *CSD2* mRNA levels for catalyzing the dismutation of superoxide radicals into H_2_O_2_ and sink Cu. On the contrary, decreased *CSD1* and *CSD2* transcripts abundance allows efficient delivery of limited Cu to PC, which is essential for photosynthesis when Cu is limited [60], [61]. It is likely with the concentration of Cu limited, excess Cd induces miR398 expression, which inhibits the function of Cu/Zn/SOD (CSD) and further induces ROS accumulation [4]. In this study, miRNA398a showed up-regulation in response to Cd treatment in HX3 and ZH24 (Table 1). Glyma03g40280.2 (coding CSD1), one target of miRNA398a (Table S5), was identified to be down-regulated in two cultivars and the decreasing trend in HX3 was more obvious than that in ZH24 (data not shown). This result may imply HX3 could save more Cu for photosynthesis and a higher tolerance than that of ZH24 when exposed to Cd. In addition, miRNA398c, one homologues member of miRNA398a, was confirmed to be up-regulated in two cultivars by qRT-PCR. Glyma06g19680.1 and Glyma14g39910.1, two targets of miRNA398c, was confirmed to be down-regulated in HX3 and ZH24 (Figures 4 and 5). Glyma06g19680.1 encodes zinc finger transcription factor protein, which plays an important role in stress tolerance [62]. As shown in Table S7, the expression of Glyma06g19680.1, however, was down-regulated in the two cultivars (Figures 4 and 5), and in HX3 its expression was higher than that in ZH24 under Cd treatment. Glyma14g39910.1 encodes Prolyl oligopeptidase (prolyl endopeptidase), an atypical serine protease, which hydrolyses peptides and peptide homones after proline in peptides up to around 30 residues long [63]. When suffer from Cd exposure, the expression of Glyma14g39910.1 decreased sharply in HX3 in comparing with that in ZH24 (Figures 4 and 5). This could help alleviate the harmful effect caused by the hydrolysis of protease.

MiRNA397a and miRNA408 are two other important Cd-responsive miRNAs, which are also regulated by SPL7. Glyma18g42520.1encodes *laccase*, which have been provided involved in the lignification of *A. thaliana* stem with the *lac17* mutant [64]. Several studies reported on an increased lignin synthesis upon metal treatment [65], [66], and lignification is one of defense mechanism under Cd exposure in soybean root [67], [68]. It is well known that lignin provides structural support, and contributes to plant defense mechanism against both biotic and abiotic stresses [69]. As shown in Table S7, Glyma18g42520.1, down-regulating by miRNA397a in two cultivars (Figures 4 and 5), showed higher expression level in ZH24 than that in HX3 under Cd stress. This result seems inconsistent with that HX3 which possess a higher Cd tolerance than ZH24. As one kind of copper protein, LAC should be considered. And Cd treatment may lead to Cu deficiency, which have been elucidated by Chmielnicka and Sowa [70] in rats. So, even higher expression level of *laccase* in ZH24 with Cd treatment, compared with that in HX3 with Cd treatment, might not form functional protein when it encounters Cu deficiency caused by Cd exposure. This may explain why HX3 possess a higher tolerance. In addition, miRNA408 was identified to cleaved *cupredoxin*, *laccase* and *plantacynin* by degradome sequencing. Glyma03260.2 and Glyma08g13510.1, encoding cupredoxin and planacynin (another two copper protein), separately, showed almost the same expression patterns in four mixture samples with that of *laccase*, though the expression of Glyma03260.2 showed lower in ZH24 than that in HX3 with Cd treatment (Figures 4, 5 and Table S7).

One of the primary symptoms of Cd toxicity is root growth inhibition, with the root apex being the most sensitive part of the root [71]–[73]. Several conserved miRNAs, such as miR160, miR164, miR167, miR390 and miR396, have been well characterized playing functional roles in root development [74]–[78]. Glyma15g19460 and Glyma17g35090.1, cleaved by miR396b-5p, encode for growth-regulating factor (GRF), which is known to act in a functionally redundant fashion to positively control cell proliferation and size in leaves in *A. thaliana* [79]–[81]. Consistent with the fact that miR396 acts as a negative regulator of GRF gene expression, overexpression of miR396 negatively impacted cell proliferation in leaves and meristem size [78], [82]. The miR396b-5p, Glyma15g19460.1 and Glyma17g35090.1 were down-regulated in two cultivars (Figures 1, 4, 5, S1 and S2). This deviation may be the result of a less dominant regulation by miRNAs and the involvement of other regulations, or of dependent of the balance between the transcription rate and the post-transcriptional regulation of the target when a certain stress stimulates the transcription of a miRNA and its target. However, the expression levels of Glyma15g19460.1 and Glyma17g35090.1 in ZH24 were lower than that in HX3 with or without Cd treatment (Tables S6 and S7). This interesting result may explain why the primary root of HX3 was longer than that of ZH24 (data not shown).

Other conserved gma-miR390a-5p, which identified silencing TAS3 (*trans*-acting siRNA) in rice or *Medicago* with Cd [25], Hg [40] and Al [39], performed over 2 fold down-regulation in ZH24 and less than 1.1 fold up-regulation in HX3 (Table 1). The down-regulation of miR390 would lead the accumulation of intact TAS3 transcript and the decrease of tasiARFs and ARFs resulting in the inhibition of lateral root growth [40], [83]. ARFs (auxin response factors) play critical roles in lateral root development [84]. This result might explain the growth inhibition in ZH24 was serious and sensitive to Cd exposure.

As one novel soybean Cd-responsive miRNA, miR1535b was identified to cleave Glyma07g38620.1. And Glyma07g38620.1 encoding isopentyl transferase (IPT), which catalyses the rate-limiting first step in de novo cytokinin (CK) biosynthesis and promotes the formation of isopentenyladenosine-5′-monophosphate (iPa) [85], [86]. CK has been known to inhibit primary root elongation in *A. thaliana* [87], and overexpression of *ipt* in leaves and roots can promote stress tolerance in *Agrostis stolonifera* [88]. In our study, Glyma07g38620.1 displayed an apparent up-regulation in HX3 and slight down-regulation in ZH24 under Cd exposure (Table S8), so does the CK, which may explain why HX3 possessed higher tolerance and distinctly primary root elongation inhibition than that of ZH24 under Cd stress. In addition, ARFs were up-regulated in HX3 and down-regulated in ZH24 due to the regulation of gma-miR390a-5p. CK has been known to inhibit primary root elongation and suggested to act as an auxin antagonist in the regulation of lateral formation. And the up-regulation of ARFs might lead to the promotion of lateral root growth. But in what rats of ARFs and CK it regulated the growth of root and stress tolerance in soybean under Cd exposure need further study.

## Supporting Information

Figure S1 **The relative expression levels of 16 Cd-responsive miRNAs between HX3-CK and HX3-Cd in the root of soybean by qRT-PCR.** The expression levels of miRNAs were normalized to the level of F-box. The french grey shading bar represents the relative expression level of miRNAs in HX3-CK. The dark grey shading bar represents the relative expression level of miRNAs in HX3-Cd. The results are averages ± SD of the duplicates of three biological replicates. Significance of the changes between HX3-CK and HX3-Cd was checked with Student's *t*-test at the level of 0.01<*P*≤0.05 (shown as “*”) and *P*≤0.01 (shown as “**”).
(TIF)

Figure S2 **The relative expression levels of 16 Cd-responsive miRNAs between ZH24-CK and ZH24-Cd in the root of soybean by qRT-PCR.** The expression levels of miRNAs were normalized to the level of F-box. The french grey shading bar represents the relative expression level of miRNAs in ZH24-CK. The dark grey shading bar represents the relative expression level of miRNAs in ZH24-Cd. The results are averages ± SD of the duplicates of three biological replicates. Significance of the changes between ZH24-CK and ZH24-Cd was checked with Student's *t*-test at the level of 0.01<*P*≤0.05 (shown as “*”) and *P*≤0.01 (shown as “**”).
(TIF)

Table S1 **The sequence of 953 probes used for microarray hybridization experiments.**
(DOC)

Table S2 **The stem–loop RT and qRT-PCR primer sequences for miRNAs expression analysis.**
(DOC)

Table S3 **The primer sequences for qRT-PCR analysis of target genes.**
(DOC)

Table S4 **The miRNAs from other species show strong signals (signal >1000) in soybean.**
(DOC)

Table S5 **Predicted targets of miRNAs identified by degradome sequencing in soybean root.**
(XLSX)

Table S6 **Expression profiles for ten target genes analysed in HX3-CK and ZH24-CK.**
(DOC)

Table S7 **Expression profiles for ten target genes analysed in HX3-Cd and ZH24-Cd.**
(DOC)

Table S8 **The relative expression level of Glyma07g38620.1 by qRT-PCR.**
(XLS)
