# Supplementary figures and images for: Identification and Comparative Analysis of Cadmium Tolerance-Associated miRNAs and Their Targets in Two Soybean Genotypes
Source: PLoS One. 2013 Dec 10;8(12):e81471. doi: 10.1371/journal.pone.0081471 (PMC3867309; doi:10.1371/journal.pone.0081471)

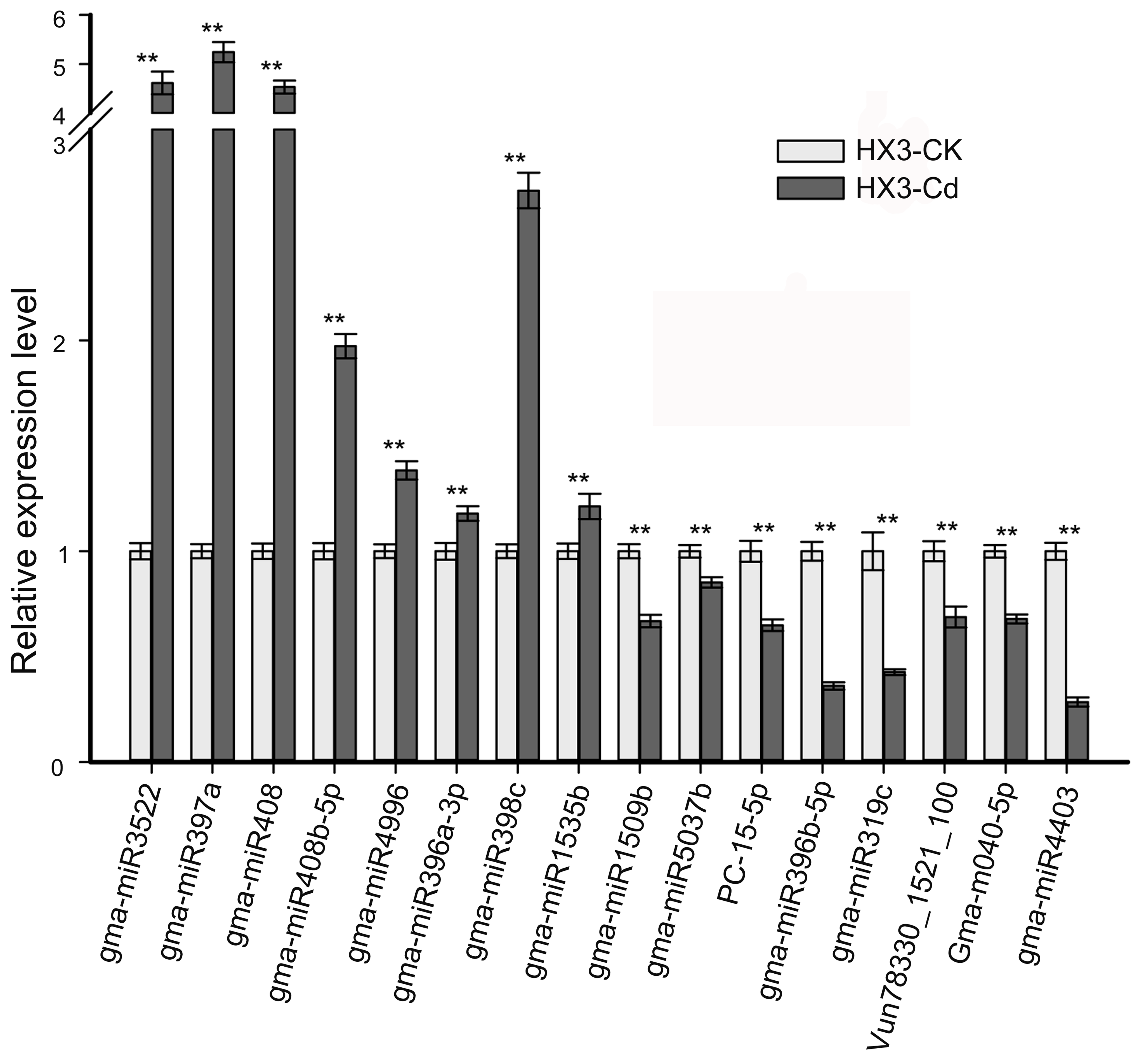

Supplement: Figure S1 — The relative expression levels of 16 Cd-responsive miRNAs between HX3-CK and HX3-Cd in the root of soybean by qRT-PCR. The expression levels of miRNAs were normalized to the level of F-box. The french grey shading bar represents the relative expression level of miRNAs in HX3-CK. The dark grey shading bar represents the relative expression level of miRNAs in HX3-Cd. The results are averages ± SD of the duplicates of three biological replicates. Significance of the changes between HX3-CK and HX3-Cd was checked with Student's t-test at the level of 0.01<P≤0.05 (shown as “*”) and P≤0.01 (shown as “**”). (TIF) [file pone.0081471.s001.tif]

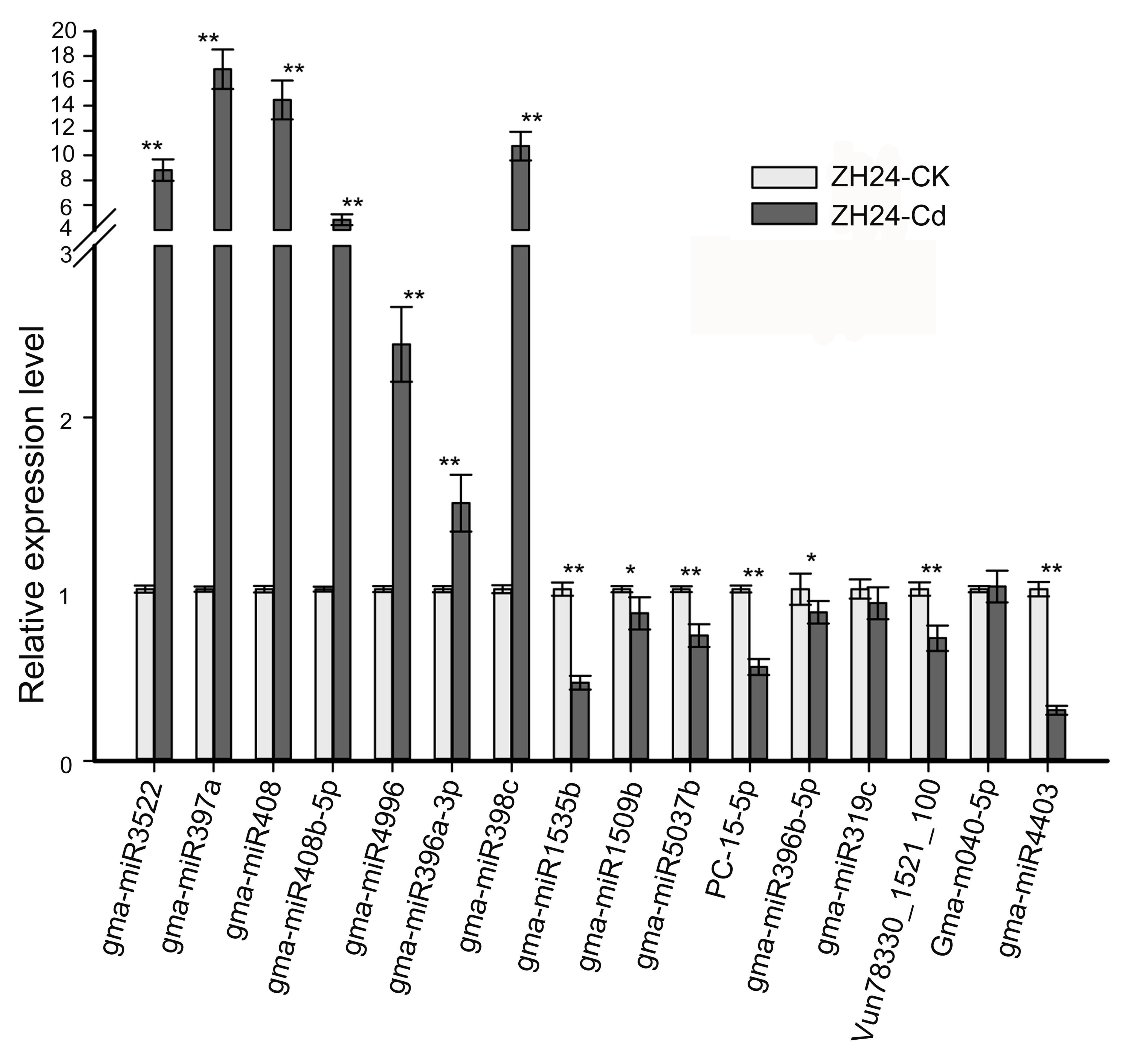

Supplement: Figure S2 — The relative expression levels of 16 Cd-responsive miRNAs between ZH24-CK and ZH24-Cd in the root of soybean by qRT-PCR. The expression levels of miRNAs were normalized to the level of F-box. The french grey shading bar represents the relative expression level of miRNAs in ZH24-CK. The dark grey shading bar represents the relative expression level of miRNAs in ZH24-Cd. The results are averages ± SD of the duplicates of three biological replicates. Significance of the changes between ZH24-CK and ZH24-Cd was checked with Student's t-test at the level of 0.01<P≤0.05 (shown as “*”) and P≤0.01 (shown as “**”). (TIF) [file pone.0081471.s002.tif]
