# Supplementary material for: Identification and Comparative Analysis of Cadmium Tolerance-Associated miRNAs and Their Targets in Two Soybean Genotypes
Source: PLoS One. 2013 Dec 10;8(12):e81471. doi: 10.1371/journal.pone.0081471 (PMC3867309; doi:10.1371/journal.pone.0081471)
Supplement: Table S1 — The sequence of 953 probes used for microarray hybridization experiments. (DOC) [file pone.0081471.s003.doc]

**Table S1. The sequence of 953 probes used for microarray hybridization experiments.**

| 259 unique custome miRNAs published in papers | | |
| --- | --- | --- |
| Index | miR_name | miR_seq |
| 1 | Vun78330_1521_100 | TTTGGACTGAAGGGAGCTCCT |
| 2 | Wgs161461_230_140 | TTCCAAAGGGATCGCATTGATC |
| 3 | Wgs154295_330_120 | TGAAGTGTTTGGGGGAACTCT |
| 4 | Gma-m006-3p | CCTCATTCCAAACATCATCTAA |
| 5 | Gma-m006-5p | AGAGGTGTTTGGGATGAGAGA |
| 6 | Gma-m014-3p | GCTGGATGTCTTTGAAGGAAT |
| 7 | Gma-m014-5p | TCCCTCAAAGGCTTCCAGTAT |
| 8 | Gma-m018-5p | GGAATGTCGTTTGGTTCGAGA |
| 9 | Gma-m007-5p | GGAATGGGCTGATTGGGAAGT |
| 10 | Gma-m017-5p | CCTCAAAGGCTTCCACTACTG |
| 11 | vun_cand026 | GGTGAGTAAATTTGGAAGGGAAGA |
| 12 | vun_cand027 | CGTTGGGCCTGTCTAGATCA |
| 13 | vun_cand028 | TATGTTGTTTCTTTATTGGTA |
| 14 | vun_cand029 | AGAAAGAGTGAGGGAATGTGA |
| 15 | vun_cand030 | TCTTTCTTCTTTGGCTCTCGTGAT |
| 16 | vun_cand031 | GGGGATGTAGGTCAGATGGTA |
| 17 | vun_cand032 | TGAAGTCGTGCCATTATGGCAAGA |
| 18 | vun_cand033 | AGAATTGTGCACCTCTGTCAGGTC |
| 19 | vun_cand034 | TAAGAGAATGATATCTTAGTCATT |
| 20 | vun_cand035 | TGGCCATACTTTGTAGAGTTGC |
| 21 | vun_cand036 | TCAGAGGAAACAACACTTGTAC |
| 22 | vun_cand037 | TCCGACACACATTTTTGGATG |
| 23 | vun_cand038 | TGATAAATATATGATTGTTATTGT |
| 24 | vun_cand039 | TTATAGTGAAAGTGCAGAAGA |
| 25 | vun_cand040 | GGAGTGAATCTGAGAACACAAG |
| 26 | vun_cand041 | ACACAAGTCCATCAGAGCTGC |
| 27 | vun_cand042 | CGGCTTGTAAGATGAGGTTTGC |
| 28 | vun_cand043 | AGGCAATTTTTAGCATTCAATGAA |
| 29 | vun_cand044 | TTAGTGATAAAAAATTGTTAGT |
| 30 | vun_cand045 | CGTGCTGAGAAAGTTGCTTCT |
| 31 | vun_cand046 | AGGTGCAGGTGCATCTGCAGG |
| 32 | vun_cand047 | TCTCGGAAATCTTGTTAGCCT |
| 33 | vun_cand048 | TGGTCTCTAAACTTTAGAAATGAA |
| 34 | vun_cand049 | CTAGGAAGTCCTTTCTTCTTT |
| 35 | vun_cand050 | TATTTAGGTGTAGTGATTTGATGT |
| 36 | vun_cand051 | TTTGATCATATGAAAAAAGTTT |
| 37 | vun_cand052 | CGAGAGCCACTCGCCTAAGCGA |
| 38 | vun_cand053 | GTAATTGAGTTAAAAGGACTATAT |
| 39 | vun_cand054 | AGCAAGTTGAGGATGGAGCTT |
| 40 | vun_cand055 | CCACTGTAGTAGCTCTCGCTCA |
| 41 | vun_cand056 | TTGTATAGAGATTAATGAAGA |
| 42 | vun_cand057 | TGTCGCAGGAGAGATGACATTG |
| 43 | vun_cand058 | TTAAGCAGAATGATCAAATTG |
| 44 | vun_cand059 | TTCAGTGTCATTGCCATTCTC |
| 45 | vun_cand061 | CATTCATTTTTAAAGTTTAGGGAC |
| 46 | vun_cand062 | CTCCTAGGGTCTGGTTTGGAAA |
| 47 | miR2911 | CGGTGGACTGCTCGAGCTGCT |
| 48 | Wgs76823_264_120 | GACAGAAGAGAGTGAGCAC |
| 49 | Vun278580_192_200 | ATTGGAGTGAAGGGAGCACCA |
| 50 | Wgs126513_129_120 | TCGATAAACCTCTGCATCCGG |
| 51 | Wgs220873_156_100 | TGAAGCTGCCAGCATGATCTTA |
| 52 | Vun54590_44_120 | TGAGCCAAGGATGACTTGCCGG |
| 53 | Wgs43305_125_120 | TGAGCAGGGATGGCTTGCCGG |
| 54 | Vun278048_162_120 | TTGAGCCGCGTCAATATCTTA |
| 55 | Vun37466_339_240 | TTGAGCCGTGCCAATATCACGA |
| 56 | Vun99012_489_100 | TATTGGTCCGGTTCAATGAGA |
| 57 | Wgs106389_241_120 | TTGAGCCGCGTCAATATCTCA |
| 58 | Wgs208270_415_120 | TGAGAATCTTGATGATGCTGC |
| 59 | Wgs218543_62_100 | TAATCTGTATCCTGAGGTTTA |
| 60 | Wgs45118_98_100 | TAATCTGCATCCTGAGGTGTA |
| 61 | Est17093_129_220 | TGGACTGAAGGGAGCTCCTTC |
| 62 | Est17179_297_240 | TTGGACTGAAGGGGCCTCTTC |
| 63 | Wgs166059_376_200 | TTGGACTGAAGGAGCCCCTTC |
| 64 | Vun277940_0_100 | TCCAAAGGGATCGCATTGATCC |
| 65 | Vun101791_746_100 | TTTGGCATTCTGTCCACCTCC |
| 66 | Vun36607_207_140 | TGAAGTGTTTGGGGGAACTTT |
| 67 | Vun278320_159_120 | TACGCAGGAGAGATGACGCTG |
| 68 | GSS51217_86_100 | GGAATGGGCTGATTGGGAAGC |
| 69 | Wgs223356_53_140 | TGCATTTGCACCTGCGCTTTG |
| 70 | Vun272231_288_100 | TCTTGCTCAAATGAGTATTCCA |
| 71 | Gma-m001-5p | CTGACAGAAGATAGAGAGCAC |
| 72 | Gma-m002-5p | CAGGGGAACAGGCAGAGCATG |
| 73 | Gma-m003-5p | TCCATTGTCGTCCAGCGGTTA |
| 74 | Gma-m004-5p | TGGGTGAGAGAAACGCGTATC |
| 75 | Gma-m004-3p | TACGGGTCGCTCTCACCTAGG |
| 76 | Gma-m005-5p | AGCCAAGAATGACTTGCCGGAA |
| 77 | Gma-m005-3p | CGGGCAAGTTGTTTTTGGCTAC |
| 78 | Gma-m008-5p | CTGGGTGAGAGAAACACGTAT |
| 79 | Gma-m008-3p | ACGGGTCGCTCTCACCTGGAG |
| 80 | Gma-m009-3p | TATTGACGCTGCACTCAATCA |
| 81 | Gma-m010-3p | CGAGCCGAATCAATACCACTC |
| 82 | Gma-m012-5p | TCACGCCTAATCACTGACGCAT |
| 83 | Gma-m013-5p | CGTGACTTGAACAGCTACGTA |
| 84 | Gma-m015-5p | TTATCAGTAGCATCATCATCA |
| 85 | Gma-m016-5p | TGTTGGACGGTTCAATCAAA |
| 86 | Gma-m017-3p | GTAGTGGATGCCTAGAGGTC |
| 87 | Gma-m020-3p | TTATGGAAGAAGCTCAATTCA |
| 88 | Gma-m022-3p | TCATTGAGTGTAGCATTGATG |
| 89 | Gma-m024-5p | TGACAGAAGAGAGTGAGCACT |
| 90 | Gma-m026-5p | TGAGCCAAGGATGACTTGCCG |
| 91 | Gma-m027-3p | GCGAGACATCTTGGCTCACT |
| 92 | Gma-m029-3p | AAAAGCACTTAAGGAACGGTA |
| 93 | Gma-m035-3p | GCGAGACATCTTGGTTCATT |
| 94 | Gma-m039-3p | TCGGACCAGGCTTCATTCCCCA |
| 95 | Gma-m040-5p | AAGCTCAGGAGGGATAGCACCA |
| 96 | Gma-m041-3p | GCGAGACATCTTGGCTCATT |
| 97 | Gmam0044-3p | TCAATCCTGGAAGAACCGGCG |
| 98 | Gmam0047-5p | TGAGCCAGGATGGCTTGCCGGC |
| 99 | Gmam0047-3p | TGGTGGCCATCCATGCTCATA |
| 100 | Gmam0048-3p | TCGACACTGCACTCAATCATG |
| 101 | PN-miR4346_L-1R+1 | AAAGACCAAACGAGAAGCTGCATA |
| 102 | PN-mir4400-p3 | CGAAGAACTCCAGAATACGTTC |
| 103 | PN-miR5139_L-1 | AACCTGGCTCTGATACCA |
| 104 | PN-miR4370_L-2R+2 | TAGACTCGTCCGATTTTGCGTAGA |
| 105 | PC-36-5p | GAGTTGGCGAGTTGGACACGTGGC |
| 106 | PN-miR444f | TGCAGTTGTTGCCTCAAGCTT |
| 107 | PN-mir4413-p3 | TACAGTGACTTACAATTCTCT |
| 108 | PN-mir156g-p3 | TTTTGTGCTCTCTATCTTCT |
| 109 | PC-42-5p | AAGAAACGTTGACTCTCCGTGTT |
| 110 | PC-29-3p | CTAGTCCTTGGGATGCAGATTACG |
| 111 | gso-mir1510a-p5 | AGGGATAGGTAAAACAATGAC |
| 112 | PN-miR2911_L-3_1ss15CT | CGGGGGACGGGTTGGGA |
| 113 | PC-20-3p | CAACGGTCATAATGTAGATTTA |
| 114 | PN-mir398b-p5 | GAGTGGATCTGAGAACACAAGG |
| 115 | PN-mir2107-p3 | ACGATCCACTCCTACTCAGGTTCA |
| 116 | PC-31-5p | TGAAGGTACAAACGATGTAGAATG |
| 117 | PC-18-3p | CATAGAATAGATAGGTACATAT |
| 118 | PC-37-5p | TACAAACGATGTAGAATGTGGACA |
| 119 | PC-33-3p | GGAGAACAAAGAAGCAGCTAAATTC |
| 120 | PC-43-5p | ATCGGGATGCTCAGTTCGCATGGT |
| 121 | PN-miR1520d_L+1R+1 | AATCAGAACATGACACGTGACAAT |
| 122 | PC-56-3p | GGCGAGGAATCTGGGCTCATT |
| 123 | PN-mir156e-p3 | GCTCTCTAGTCTTCTGTCATC |
| 124 | PC-62-3p | TGGGGCTTGATCCAAGATAGG |
| 125 | PC-61-5p | AACTTACTGACTCGTTGACTCGGT |
| 126 | PN-miR1523_L+1 | TATGGGATAAATGTGAGCTCA |
| 127 | PN-miR157d | TGACAGAAGATAGAGAGCAC |
| 128 | PN-miR5072_L-4 | TCCCCAGCGGAGTCGCCA |
| 129 | PN-miR4343a_1ss19GA | AAAAAACTTACGGATCAAATTGAT |
| 130 | PC-38-5p | TCTACGAGTCGAGTTCATGGAACT |
| 131 | PN-miR166a*_R+1 | GGAATGTTGTCTGGCTCGAGGT |
| 132 | PN-mir156f-p3 | GCTCTCTCTTCCTCTGTCATC |
| 133 | PN-mir1517-p3 | ACATCGGGTAGGTTCTATTGGT |
| 134 | PC-7-5p | TTTACAGGGAACCACTCAGACGTT |
| 135 | PC-60-3p | TTTCCCGGCAATGGAACCA |
| 136 | PC-41-3p | AAATCAGATGATATGGACTTAAAT |
| 137 | PC-55-3p | GCTCACTTCTCTTTCTGTCAAC |
| 138 | PC-10-5p | AACGTTGACTGTTAACCTCAGACG |
| 139 | PC-17-3p | TGACCGGAACTATTTGACTGTT |
| 140 | PN-mir1520j-p5 | ACTCCATGAAAGATGAAATGCATT |
| 141 | PN-mir4360-p3 | TTGTCAATCCGTACGTACGTCAAC |
| 142 | PN-mir319a-p5 | TAGCTGCCGACTCATTCATCCA |
| 143 | PC-35-5p | AAAAGGACACATGACTCACACCTA |
| 144 | PN-miR1511_R-2 | AACCAGGCTCTGATACCA |
| 145 | PN-mir2119-p5_5ss10AT18TG21CG22TA23CG | ATTTTTTATTCTTTAATGTCGAGT |
| 146 | gso-mir1508a-p5 | ACTGCTATTCCCATTTCTAAA |
| 147 | PN-miR1514b_L+3R-2 | ATTTTCATTTTTAAAATAGACA |
| 148 | PN-miR397a_L-1 | CATTGAGTGCAGCGTTGATG |
| 149 | PN-mir4364a-p5_1ss3CT | TGTCATCTTGCGGAAGAACTTCT |
| 150 | gso-mir2109-p3 | GGAGGCGTAGATACTCACACC |
| 151 | PC-21-5p | CAAAGATTCATCGTAGGCTAGACT |
| 152 | PC-11-5p | GAGTTAAATCGGAAGTAGAATCGA |
| 153 | PN-miR394a_R+1 | AGCTCTGTTGGCTACACTTTG |
| 154 | PN-miR1520j_L-1R+1 | AGAACGTGACACATGACAATCAAC |
| 155 | PC-27-3p | AGTCAACGTCTGAGATTAACGGTC |
| 156 | PN-mir1520l-p5 | ATTGGTCAATGAAAACAACAATGC |
| 157 | PN-miR2111a_R-1 | TAATCTGCATCCTGAGGTTT |
| 158 | PC-51-3p | ACTGAAAACACTGAAAACGAAAAT |
| 159 | PC-65a-3p | TTCGAATGGATACTGATACATCTG |
| 160 | PC-24-5p | CTATGGGAGCTGGCCATGCCCGAAG |
| 161 | PN-miR1513_L+2R-2 | TATGAGAGAAAGCCATGACTT |
| 162 | PN-mir4406-p3 | TCGTCTCAGAACCGATGTAGAATG |
| 163 | PN-mir4383-p5 | TCAACTGAGATCCGGTACCTAACC |
| 164 | PN-mir4373-p3 | GGCCATACAGATTGTCAATCCGTA |
| 165 | PN-mir1523-p3 | CGAGCTCACATTTATTTCAAAA |
| 166 | PN-miR4366_L-1R+1 | TACTTAGTAGAGATTTGTTGGT |
| 167 | gso-mir167a-p3 | GATCATGTGGCTGCTTCACC |
| 168 | PN-miR4360_L-1R+1 | AGTTGACGTACGTACGGATTGACA |
| 169 | PC-28-3p | CATTTGGGCACCTATTTTGACTC |
| 170 | PN-mir1520i-p5 | ACTGTCACGTGTCATGTTCTGATT |
| 171 | PN-mir162-p5_1ss5TG | TGGAGGCAGCGGTTCATCGATC |
| 172 | gso-mir1509a-p3 | ACCGTGTTTCCTTGGTTAACG |
| 173 | PN-mir1509b-p3 | ACTGTGTTTCCTTGGTTAAAG |
| 174 | PC-5-5p | GCATATTAGCAATGATGATCTG |
| 175 | PC-19-5p | TCACGCCTAATCACTGACGCA |
| 176 | PN-mir4371b-p3 | TGCCACATCATCACCTAACGGAAG |
| 177 | PN-miR4413_L+1 | TAAGAGAATTGTAAGTCACTG |
| 178 | PC-59-3p | CAAAATGATCATCGACTTTACATT |
| 179 | PC-5-3p | CAATGATGATCTGATAGTTTTT |
| 180 | PN-miR160c_1ss15AC | TGCCTGGCTCCCTGCATGCCA |
| 181 | PN-miR4352b_L-1R+1 | AAAATGTAGACATTCTAAGACGGT |
| 182 | PN-miR894_L-3R+1 | TTCACGTCGGGTTCACCA |
| 183 | PC-34-5p | GCTGGTTTGCCCGAGA |
| 184 | gso-mir3522a-p3 | AGCTGCTCATCTGTTCTCAGG |
| 185 | PC-46-5p | CTATATGATGAAGATA |
| 186 | PC-12-5p | AAATCTACATGCGGATCAAGTTGA |
| 187 | PN-miR172e | GGAATCTTGATGATGCTGCAT |
| 188 | PC-22-5p | CAAGGAATTTTAGAAGATAAGACA |
| 189 | PN-miR164a_L-1 | GGAGAAGCAGGGCACGTGCA |
| 190 | PC-1-3p | GTCTCTGATGAATGCTCAAATTCT |
| 191 | PN-miR1509b_R+1 | TTAATCAAGGAAATCACGGTTG |
| 192 | PC-52-5p | TGAGGGCAAAGATATTAGAGAA |
| 193 | PC-8-5p | ATTGTTGAGACGATTCTGTAGACG |
| 194 | PC-57-3p | GCATACAGGGAGTCAAGCAGA |
| 195 | PN-mir4345-p3 | ATCTTTTTAAGTTTCGTCTTA |
| 196 | PN-miR1516_L+2R-2 | CTCAAAAGAGCTTATGGCTTG |
| 197 | PC-30-3p | AAGGCTTACGGATCAAGTTGGTAT |
| 198 | PN-miR1520i_1ss7AT | AACGTGTCACGTGACGGTCAACAT |
| 199 | PC-67a-3p | AATTAACTTACGGATCAACTTGAT |
| 200 | PN-miR1514a_R+3 | TTCATTTTTAAAATAGGCATTGGG |
| 201 | PC-63-3p | GTAGTGGATGCCTAGAGGTCC |
| 202 | gso-mir2218-p5 | GGAGATGGGAGGGTCGGTAAAG |
| 203 | PN-miR1520l_L+1R-1 | CAATCAGAACATGACACGTGATAG |
| 204 | PN-mir398a-p5 | CGGAGGAGTGAATCTGAGAACAC |
| 205 | PN-miR159c_R-3 | ATTGGAGTGAAGGGAGCT |
| 206 | PC-25-3p | CTACATAAGGCACGAGATCATC |
| 207 | PN-mir4347-p3 | AGAAGCTTGCGGATCAATTTGATC |
| 208 | PN-mir159c-p5 | CAAGTTGGAGCTCTCTACACTC |
| 209 | PN-miR172b* | GCAGCACCATCAAGATTCACA |
| 210 | PN-miR4371b_L-1R+1 | AGTGATGACGTGGTAGACGGAGTG |
| 211 | PN-miR166_L-1 | CGGACCAGGCTTCATTCCCC |
| 212 | PC-4-5p | GTCGTTGTAGTATAGTGGTAAGT |
| 213 | PN-miR1520m_L+2R-2 | TCAATCAGAACATGACATGTGACA |
| 214 | PN-miR4378b_R+1 | TAGAACTGTCTTAGAATGTGCTACA |
| 215 | PC-32-3p | TATATTCGGATATTCACATT |
| 216 | PC-64-5p | AATGAAAACGCGGTGACAAAGAAT |
| 217 | PN-mir4369-p5 | GATCAACTTGATCCGGAAGAGACT |
| 218 | PC-40-3p | AATACGTAAGGCTTGAGCTTGACT |
| 219 | PN-mir1520m-p5 | ATTGGATGATGACTGTTACGTGCC |
| 220 | PN-mir4358-p5 | TTTAGGTTGCGTCAGAAAGTAATT |
| 221 | PC-16-5p | CTCTCCCTCAAGGGCTTCTCG |
| 222 | PC-49-3p | AAAGCCATGACTTACACACGC |
| 223 | PN-mir390b-p3 | CGCTATCTATCTTGAGCTTCA |
| 224 | PC-39-5p | TAGATTTTAAAGTTGCGGATCA |
| 225 | PC-44-3p | ATGAACCCTTTGAGATCACTGGTT |
| 226 | gso-mir1507a-p5 | AGAGGTGTATGGAGTGAGAGA |
| 227 | PN-mir156d-p3 | GCTCTCTATACTTCTGTCATC |
| 228 | PC-50-5p | GATTAAGATGCACATACCTCTGAT |
| 229 | PC-47-3p | CGTTGTAGTATAGTGGTAAGTATT |
| 230 | PC-9-5p | TTCCCCACAGACGGCGCCA |
| 231 | PN-mir1515-p3_1ss7CT | TGATCATTCACGCAAAATGA |
| 232 | PN-miR4372_L+1R-1 | ATAAAATCGTGACATGTGACGGTC |
| 233 | PN-miR4406_L+2R-2 | ATATTGATTCTGAGAGAACCGGTG |
| 234 | gso-mir1510b-p5 | AGGGATAGGTAAAACAACTAC |
| 235 | PC-48-3p | TTAGCTTCTTTCACCTTTCCC |
| 236 | PN-miR2107_R+3 | CAAACCTCCGTAGCCTGTATCAAA |
| 237 | gso-mir1507b-p5 | AGAGATGTATGGAGTGAGAGA |
| 238 | PC-15-5p | TCCGTTGTAGTCTAGTTGGTTAGGA |
| 239 | PC-45-5p | AGACGGTAAGAAGAGAATTTCAAT |
| 240 | PC-1-5p | TGGCCTGTGATGCATCAATTTGTC |
| 241 | PN-miR4374a_L+2 | TCTAAGACGGTCGTGATGTCAGCA |
| 242 | PN-mir1516-p5 | TTGGATACAAGTTATAAGCTCT |
| 243 | PN-miR4399_L-2R+4 | AACGAAAAAGGACTAACGACAGGT |
| 244 | PC-58-5p | TTAGTTGAATGGTACTGTAGTAGT |
| 245 | gso-mir482a-p5 | GGGAAGGCATGGGTATGGGGG |
| 246 | PN-miR4404_L+1R-1 | AATTCGTGGAAGACTGGCGGGTCA |
| 247 | PC-53-5p | TCAGTCTCGGTGTGGATACACGTA |
| 248 | PN-mir4399-p5 | AAGTAATGACGTGGGAGACAGAGT |
| 249 | PN-miR4364a_L-2R+2 | CGAGATCGCACGGAAGAAGGTTCT |
| 250 | PC-4-3p | GTCGTTGTAGTATAGTGGTAAGTA |
| 251 | PN-mir1511-p5 | GTGGTATCAGGTCCTGCTTCA |
| 252 | PN-miR4345_L+4R-2 | AAGCTAAGACGGAACTTACAAAGA |
| 253 | PN-mir169c-p3_1ss14CT | GGCAGGTCATCCTTTGGCTATA |
| 254 | PN-miR4393b_L+1R-1 | CTTGAAAAGGGACAGCAGAGAAGC |
| 255 | PN-miR166b_2ss20TC21AT | TCGGACCAGGCTTCATTCCCT |
| 256 | PC-26-3p | CATCGGTCGAGAGCGTTCTT |
| 257 | PN-mir4343a-p3 | AAAGGCTTACGAATCAACTTGATC |
| 258 | gso-mir482b-p5_1ss5GA | TATGAGGGGATTGGGAAGGAA |
| 259 | PC-54-5p | TCACAAAGGGAACGAGGACTCC |
| 694 miRNAs of legumes in miRBase 18.0 | | |
| Index1 | miR_name | miR_seq |
| 1 | gma-miR390b | AAGCTCAGGAGGGATAGCACC |
| 2 | gma-miR164 | TGGAGAAGCAGGGCACGTGCA |
| 3 | gma-miR172c | GGAATCTTGATGATGCTGCAG |
| 4 | gma-miR397a | TCATTGAGTGCAGCGTTGATG |
| 5 | gso-miR2218 | TTGCCGATTCCACCCATTCCTA |
| 6 | gma-miR390a-5p | AAGCTCAGGAGGGATAGCGCC |
| 7 | gma-miR1507a | TCTCATTCCATACATCGTCTGA |
| 8 | gma-miR403a | TTAGATTCACGCACAAACTTG |
| 9 | gma-miR1515 | TCATTTTGCGTGCAATGATCTG |
| 10 | gma-miR2119 | TCAAAGGGAGTTGTAGGGGAA |
| 11 | ahy-miR160-5p | TGCCTGGCTCCCTGAATGCCA |
| 12 | gma-miR166a-5p | GGAATGTTGTCTGGCTCGAGG |
| 13 | gma-miR166h-3p | TCTCGGACCAGGCTTCATTCC |
| 14 | gma-miR167g | TGAAGCTGCCAGCATGATCTGA |
| 15 | gma-miR169n | CAGCCAAGGGTGATTTGCCGG |
| 16 | gma-miR171e | TGATTGAGCCGTGCCAATATC |
| 17 | lja-miR2111 | TAATCTGCATCCTGAGGTTTA |
| 18 | gma-miR319a | TTGGACTGAAGGGAGCTCCC |
| 19 | gma-miR319d | TGGACTGAAGGGGAGCTCCTTC |
| 20 | gma-miR395a | CTGAAGTGTTTGGGGGAACTC |
| 21 | gma-miR396b-3p | GCTCAAGAAAGCTGTGGGAGA |
| 22 | gma-miR171k | CGATGTTGGTGAGGTTCAATC |
| 23 | gma-miR4387e | TGTTAGTGATAAGGCGTGATG |
| 24 | gma-miR5671 | CATGGAAGTGAATCGGGTGAC |
| 25 | gma-miR394a | AGCTCTGTTGGCTACACTTT |
| 26 | gma-miR2111 | GTCCTTGGGATGCAGATTACG |
| 27 | gma-miR530b | TGCATTTGCACCTGCACTTTA |
| 28 | gma-miR5674 | TAATTGTGTTGTACATTATCA |
| 29 | gma-miR5668 | AGCAATGGAATTATAGACTGC |
| 30 | gma-miR5677 | TTTGGTCTTTAATCAAGCTGA |
| 31 | gma-miR1508c | TAGAAAGGGAAATAGCAGTTG |
| 32 | gma-miR1509a | TTAATCAAGGAAATCACGGTCG |
| 33 | gma-miR1510b-3p | TGTTGTTTTACCTATTCCACC |
| 34 | gma-miR167c | TGAAGCTGCCAGCATGATCTG |
| 35 | gso-miR2109 | TGCGAGTGTCTTCGCCTCTGA |
| 36 | gso-miR3522a | TGAGACCAAATGAGCAGCTGA |
| 37 | gma-miR482b-3p | TCTTCCCTACACCTCCCATACC |
| 38 | gma-miR1520f | CAATCAGAACATGACACATGACAA |
| 39 | gma-miR1535b | CTTGTTTGTGGTGATGTCTAG |
| 40 | ahy-miR156c | TTGACAGAAGAGAGAGAGCAC |
| 41 | ahy-miR156b-5p | TTGACAGAAGATAGAGAGCAC |
| 42 | ahy-miR159 | TTTGGATTGAAGGGAGCTCTA |
| 43 | gma-miR168 | TCGCTTGGTGCAGGTCGGGAA |
| 44 | gma-miR169c | AAGCCAAGGATGACTTGCCGA |
| 45 | aau-miR319 | TTGGACTGAAGGGAGCTCCCT |
| 46 | gma-miR393 | TCCAAAGGGATCGCATTGATC |
| 47 | gma-miR396b-5p | TTCCACAGCTTTCTTGAACTT |
| 48 | gma-miR398a | TGTGTTCTCAGGTCACCCCTT |
| 49 | gma-miR4347 | AAGCTTCTTACGGATCAAGTTGAT |
| 50 | gma-miR4358 | CAGTGCATGACTATATCGCCAG |
| 51 | gma-miR4359b | AACGCGTGATATGTTAACATCGGT |
| 52 | gma-miR4365 | AAGAACTTCTTCCGCGAGATCGCA |
| 53 | gma-miR4369 | GGATCAAGCTGATCCGGAAGTGGA |
| 54 | gma-miR4373 | AAGTTGACGTACGTACGGATTGAC |
| 55 | gma-miR4380b | TATGGTCATACGGATTGTTGAT |
| 56 | gma-miR4383 | TATTGGATCTCAGTTGAACCGGTC |
| 57 | gma-miR4400 | TTCGGAAAAATTCTGGAAGACGTC |
| 58 | gma-miR4411 | TTATTGTAACTAATTTGTCGGT |
| 59 | gma-miR4415a-5p | AAGTTGTGATGAGAATCAATG |
| 60 | gma-miR4415a-3p | TTGATTCTCATCACAACATGG |
| 61 | gma-miR156a | TGACAGAAGAGAGTGAGCAC |
| 62 | gma-miR160 | TGCCTGGCTCCCTGTATGCCA |
| 63 | aau-miR162 | TCGATAAACCTCTGCATCCAG |
| 64 | gma-miR172a | AGAATCTTGATGATGCTGCAT |
| 65 | ahy-miR394 | TTGGCATTCTGTCCACCTCC |
| 66 | aau-miR396 | TTCCACAGCTTTCTTGAACTG |
| 67 | gma-miR398c | TGTGTTCTCAGGTCGCCCCTG |
| 68 | gma-miR169j-3p | TTTCGACGAGTTGTTCTTGGC |
| 69 | gma-miR169j-5p | TAGCCAAGAATGACTTGCCGG |
| 70 | gma-miR172h-5p | GCAGCAGCATCAAGATTCACA |
| 71 | mtr-miR169p | TGAGCCAGGATGGCTTGCCGG |
| 72 | mtr-miR399l | TGCCAAAGGAGAGTTGCCCTG |
| 73 | aau-miR160 | TGGCATACAGGGAGCCAGGCA |
| 74 | aau-miR172 | TGAGAATCTTGATGATGCTGCAT |
| 75 | aau-miR2086 | GACATGAATGCAGAACTGGAA |
| 76 | aau-miR168 | ATTCAGTTGATGCAAGGCGGGATC |
| 77 | ahy-miR156a | TGACAGAAGAGAGAGAGCAC |
| 78 | ahy-miR156b-3p | GCTCTCTAAGCTTCTGTCATC |
| 79 | ahy-miR160-3p | GCATGAAGGGAGTCACGCAGG |
| 80 | ahy-miR167-5p | TGAAGCTGCCAGCATGATCTT |
| 81 | ahy-miR167-3p | AGATCATGTGGCAGTTTCACC |
| 82 | ahy-miR398 | TGTGTTCTCAGGTCACCCCT |
| 83 | ahy-miR408-5p | CTGGGAACAGGCAGAGCATGA |
| 84 | ahy-miR408-3p | ATGCACTGCCTCTTCCCTGGC |
| 85 | ahy-miR3508 | TAGAGGGTCCCCATGTTCTCA |
| 86 | ahy-miR3509-5p | ATACTTGAGAGCCGTTAGATGA |
| 87 | ahy-miR3509-3p | ATCTAACGACTCTCAGATATCA |
| 88 | ahy-miR3510 | TTATACCATCTTGCGAGACTGA |
| 89 | ahy-miR3511-5p | GCCAGGGCCATGAATGCAGA |
| 90 | ahy-miR3511-3p | TGTTACTATGGCATCTGGTAA |
| 91 | ahy-miR3512 | CGCAAATGATGACAAATAGA |
| 92 | ahy-miR3513-5p | TTAATTTCTGAGTTTGTCATC |
| 93 | ahy-miR3513-3p | TTGATAAGATAGAAATTGTAT |
| 94 | ahy-miR3514-5p | AGGATTCTGTATTAACGGTGGA |
| 95 | ahy-miR3514-3p | TCACCGTTAATACAGAATCCTT |
| 96 | ahy-miR3515 | AATGTAGAAAATGAACGGTAT |
| 97 | ahy-miR3516 | GCTGGGTGATATTGACAGAAG |
| 98 | ahy-miR3517 | CTGACCACTGTGATCCCGGAA |
| 99 | ahy-miR3518 | TGACCTTTGGGGATATTCGTG |
| 100 | ahy-miR3519 | TCAATCAATGACAGCATTTCA |
| 101 | ahy-miR3520-5p | AGGTGATGGTGAATATCTTATC |
| 102 | ahy-miR3520-3p | AAGGGAGACGTTTGAATTATC |
| 103 | ahy-miR3521 | TGGTGAGTCGTATACATACTG |
| 104 | gma-miR159a-5p | GAGCTCCTTGAAGTCCAATTG |
| 105 | gma-miR166a-3p | TCGGACCAGGCTTCATTCCCC |
| 106 | gma-miR167a | TGAAGCTGCCAGCATGATCTA |
| 107 | gma-miR172b-5p | GTAGCATCATCAAGATTCAC |
| 108 | gma-miR396a-3p | TTCAATAAAGCTGTGGGAAG |
| 109 | gma-miR319c | TTGGACTGAAAGGAGCTCCT |
| 110 | gma-miR156b | TGACAGAAGAGAGAGAGCACA |
| 111 | gma-miR169a | CAGCCAAGGATGACTTGCCGG |
| 112 | gma-miR159b-5p | GAGTTCCCTGCACTCCAAGTC |
| 113 | gma-miR159b-3p | ATTGGAGTGAAGGGAGCTCCA |
| 114 | gma-miR159c | ATTGGAGTGAAGGGAGCTCCG |
| 115 | gma-miR162 | TCGATAAACCTCTGCATCCA |
| 116 | gma-miR169b | CAGCCAAGGATGACTTGCCGA |
| 117 | gma-miR171a | TGAGCCGTGCCAATATCACGA |
| 118 | gma-miR390a-3p | CGCTATCCATCCTGAGTTTC |
| 119 | gma-miR390b* | TACTTGGCGCTATCTATCTTGA |
| 120 | gma-miR171b-5p | ACGGCGTGATATTGGTACGGCTC |
| 121 | gma-miR171b-3p | CGAGCCGAATCAATATCACTC |
| 122 | gma-miR482a-5p | AGAATTTGTGGGAATGGGCTGA |
| 123 | gma-miR482a-3p | TCTTCCCAATTCCGCCCATTCCTA |
| 124 | gma-miR1508a | TCTAGAAAGGGAAATAGCAGTTG |
| 125 | gma-miR1510a-5p | AGGGATAGGTAAAACAATGACTGC |
| 126 | gma-miR1510a-3p | TTGTTGTTTTACCTATTCCACCC |
| 127 | gma-miR1511 | AACCAGGCTCTGATACCATG |
| 128 | gma-miR1512 | TAACTGAAAATTCTTAAAGTAT |
| 129 | gma-miR1513 | TGAGAGAAAGCCATGACTTAC |
| 130 | gma-miR1513* | TTTAAATGTGTATAAGTCATGGT |
| 131 | gma-miR1514a | TTCATTTTTAAAATAGGCATT |
| 132 | gma-miR1514b | TTCATTTTTAAAATAGACATT |
| 133 | gma-miR1516* | CAAGTTATAAGCTCTTTTGAGAG |
| 134 | gma-miR1516 | CAAAAGAGCTTATGGCTTGTA |
| 135 | gma-miR1517 | AGTCTTGGTCAATGTCGTTCGAAA |
| 136 | gma-miR1518 | TGTGTTGTAAAGTGAATATCA |
| 137 | gma-miR1519 | TAAGTGTTGCAAAATAGTCATT |
| 138 | gma-miR1520d | ATCAGAACATGACACGTGACAA |
| 139 | gma-miR1536 | AAGCAGAGACAAATGTGTTTA |
| 140 | gma-miR1520a | TAGAACATGATACATGACAGTCA |
| 141 | gma-miR1521 | CTGTTAATGGAAAATGTTGA |
| 142 | gma-miR1522 | TTTATTGCTTAAAATGAAAT |
| 143 | gma-miR1523 | ATGGGATAAATGTGAGCTCA |
| 144 | gma-miR1524 | CGAGTCCGAGGAAGGAACTCC |
| 145 | gma-miR1525 | TGGGTTAATTAAGTTTTTAGT |
| 146 | gma-miR1520b | GTGACAGTCATCATTTAATAAGA |
| 147 | gma-miR1526 | CCGGAAGAGGAAAATTAAGCAA |
| 148 | gma-miR1527 | TAACTCAACCTTACAAAACC |
| 149 | gma-miR1528 | ATAGATTAGATCAATATATTAGT |
| 150 | gma-miR1529 | TTAAAGGAAACAATTAATCGTTA |
| 151 | gma-miR1530 | TTTTCACATAAATTAAAATAT |
| 152 | gma-miR1531 | TCGTCCATATGGGAAGACTTGTC |
| 153 | gma-miR1532 | AACACGCTAAGCGAGAGGAGCTC |
| 154 | gma-miR1520c | TTCAATAAGAACGTGACACGTGA |
| 155 | gma-miR1533 | ATAATAAAAATAATAATGA |
| 156 | gma-miR1534 | TATTTTGGGTAAATAGTCAT |
| 157 | gma-miR1535 | CTTGTTTGTGGTGATGTCT |
| 158 | gma-miR1507b | TCTCATTCCATACATCGTCTG |
| 159 | gma-miR1508b | TAGAAAGGGGAATAGCAGTTG |
| 160 | gma-miR1510b-5p | AGGGATAGGTAAAACAACTACT |
| 161 | gma-miR2107 | CAAACCTCCGTAGCCTGTATC |
| 162 | gma-miR2108a | TTAATGTGTTGTGTTTGTCGG |
| 163 | gma-miR2108b | TTAATGTGTTGTGTTTGTGAG |
| 164 | gma-miR2109 | TGCGAGTGTCTTCGCCTCTG |
| 165 | gma-miR172d | GGAATCTTGATGATGCTGCAGCAG |
| 166 | gma-miR1509b | TTAATCAAGGAAATCACGGTT |
| 167 | gma-miR4340 | TGCAGAGATAGGGACGCGCTTA |
| 168 | gma-miR4341 | TGTGTTGAAAGTTTAACATGACGG |
| 169 | gma-miR4342 | AATCGACTTAGAATGTAGGATGGT |
| 170 | gma-miR4343a | AAAAAACTTACGGATCAAGTTGAT |
| 171 | gma-miR4344 | AAGTAGACATTCTAAGACGTTGCT |
| 172 | gma-miR4345 | TAAGACGGAACTTACAAAGATT |
| 173 | gma-miR4364b | TAACAACAGCGGAAGAACCTTCTT |
| 174 | gma-miR4346 | GAAAGACCAAACGAGAAGCTGCAT |
| 175 | gma-miR4348 | AAACTTGTAAGATGGTGACATT |
| 176 | gma-miR4349 | TATTGGCTAGAGATAAGACAAAGA |
| 177 | gma-miR4350 | TCAAATGATTTTGTGTCGTTGG |
| 178 | gma-miR4351 | ATTGGGATTCAGTTGGAGTTGG |
| 179 | gma-miR4352a | ATTTCTAGGACATACTACGACGGT |
| 180 | gma-miR4353 | CAAGTCGTAGCCGGTGTTATTACT |
| 181 | gma-miR1520e | CAATAAGAACGTGACATATGACAG |
| 182 | gma-miR1520g | CAATCAGAACATGACACGTGACAA |
| 183 | gma-miR4354 | CAATTGGATCGGTCCAACCGGC |
| 184 | gma-miR4355 | CACTGTTGTGCTGGGTGTACCA |
| 185 | gma-miR4356 | CAGGACTGTCTTAGAAAGCCAGGC |
| 186 | gma-miR4357 | CAGTCGTGTGATTGTACGGTTCAT |
| 187 | gma-miR4359a | AACGAAGTGACTCTAACATCGGTT |
| 188 | gma-miR4360 | CAGTTGACGTACGTACGGATTGAC |
| 189 | gma-miR4387c | AGCGTGATGACGTGACACTCCGTC |
| 190 | gma-miR1520h | AACGTCCAATCAGAACGTGACATG |
| 191 | gma-miR4361 | CCGGAAGAGACTTACGGATCAACT |
| 192 | gma-miR1520i | AACGTGACACGTGACGGTCAACAT |
| 193 | gma-miR4362 | CCTTAGGACAGACGTCATGTAG |
| 194 | gma-miR4363 | CGATTACCAGAAGGCTTATTAG |
| 195 | gma-miR4364a | CGCGAGATCGCACGGAAGAAGGTT |
| 196 | gma-miR4380a | CGGATTGTTGATCCGTATGTGCAT |
| 197 | gma-miR396d | AAGAAAGCTGTGGGAGAATATGGC |
| 198 | gma-miR1520j | AAGAACGTGACACATGACAATCAA |
| 199 | gma-miR4366 | CTACTTAGTAGAGATTTGTTGG |
| 200 | gma-miR4367 | CTGAACCCTAGCGAAGTAAATC |
| 201 | gma-miR4368a | AAGACGGTACTTACCTCAGTAACA |
| 202 | gma-miR4371c | GACGTGACAGACGGAATATCACAT |
| 203 | gma-miR4368b | AAGGACGGTACTTACGTAAGCAAC |
| 204 | gma-miR4370 | AGTAGACTCGTCCGATTTTGCGTA |
| 205 | gma-miR4387b | AAGGTGTGATGGCATGACACTCTG |
| 206 | gma-miR4371a | AAGTGATGACATGACAAGCGAAGT |
| 207 | gma-miR4371b | AAGTGATGACGTGGTAGACGGAGT |
| 208 | gma-miR4372 | TAAAATCGTGACATGTGACGGTCA |
| 209 | gma-miR4352b | TAAAATGTAGACATTCTAAGACGG |
| 210 | gma-miR4374a | TAAGACGGTCGTGATGTCAGCA |
| 211 | gma-miR4375 | TACCACTAGTGGTCGCGCCTGGCA |
| 212 | gma-miR4376-5p | TACGCAGGAGAGATGACGCTGT |
| 213 | gma-miR4376a-3p | AGCATCATATCTCCTGCATAG |
| 214 | gma-miR4377 | TACGTCATCGCTGAATGGAAGACG |
| 215 | gma-miR4374b | TACTTTCAAAGACGTTGTTGAG |
| 216 | gma-miR4378a | ATAGGACTGTCTTAGAATGGTGTA |
| 217 | gma-miR4378b | TAGAACTGTCTTAGAATGTGCTAC |
| 218 | gma-miR4379 | TAGAGTGTATACTGTGAGAGGCCT |
| 219 | gma-miR482b-5p | TATGGGGGGATTGGGAAGGAAT |
| 220 | gma-miR4381 | TATGTGACGGTAAACGGTGACAAG |
| 221 | gma-miR4382 | TATGTTAACTGATTTCATGGAT |
| 222 | gma-miR1520k | AATCAGAACATGACACATGACAGT |
| 223 | gma-miR1520l | AATCAGAACATGACACGTGATAGT |
| 224 | gma-miR1520m | AATCAGAACATGACATGTGACAAT |
| 225 | gma-miR4384 | AATCAGACACTGCATTCAAAGACG |
| 226 | gma-miR1520n | TCAATCAGAACATGACACGTGACA |
| 227 | gma-miR1520o | TCATCGTCCAATCAGAATGTGACA |
| 228 | gma-miR4385 | AATCGATGTAGAAAAGTGATTGGT |
| 229 | gma-miR4386 | TCGAAGGTTCTGGAGAGGACTGCA |
| 230 | gma-miR4387a | AACAAGACGTGATGACGTGACACT |
| 231 | gma-miR4388 | AATCTTAGGGACCAAATTGACAGC |
| 232 | gma-miR4387d | ATGTCACTGATTAGGCATGATGAT |
| 233 | gma-miR4389 | TCGGTCGGACCGATCCAATCGGAA |
| 234 | gma-miR4390 | TCGTACTCGTCGGGTATCGGGTAT |
| 235 | gma-miR4391 | TCTCGGCAAAGAACTAAGAAGAAG |
| 236 | gma-miR4392 | TCTGCGAAAATGTGATTTCGGA |
| 237 | gma-miR4343b | TCTTACAGATCAAGTTGATTCGGA |
| 238 | gma-miR4393a | TGAGAAAAGGACGGCAGAAAAGCC |
| 239 | gma-miR4394 | AATGGACTAAAGAGAAAGGGGCCG |
| 240 | gma-miR4395 | TGGATAGGAGTATGGGCTTGAG |
| 241 | gma-miR4396 | TGTAGTTTCTAAGACGATGCTGAC |
| 242 | gma-miR4397-5p | CATCGTTGACGCTGACTGTACG |
| 243 | gma-miR4397-3p | TGTCAAAGATGTGGCGAATACT |
| 244 | gma-miR1520r | TGTCACATCCTGGTTGGACATGAA |
| 245 | gma-miR4398 | TGTCAGCGGAGTGAGAAGACGAAA |
| 246 | gma-miR4399 | TTAACGAAAAAGGACTAACGAC |
| 247 | gma-miR4393b | TTGAAAAGGGACAGCAGAGAAGCC |
| 248 | gma-miR156f | TTGACAGAAGAGAGAGAGCACA |
| 249 | gma-miR4401 | ACAACGTCTTTGAAAGTAGGCATT |
| 250 | gma-miR4402 | ACATATTATGGGTCTCAGACGGAC |
| 251 | gma-miR4403 | ACGGACACCGAACACGACACGGAC |
| 252 | gma-miR1520p | ATGTTGTTATTGGATGATGACGGT |
| 253 | gma-miR4404 | ATTCGTGGAAGACTGGCGGATCAA |
| 254 | gma-miR4405 | ATTCTAAGACGGTTATCTGGGACC |
| 255 | gma-miR4406 | ATTGATTCTGAGAGAACCGGTGTA |
| 256 | gma-miR4407 | CAGAGGAAGCAGCACTTGTACC |
| 257 | gma-miR4408 | TAACAACATTGGATGAGGGTTGGA |
| 258 | gma-miR4409 | TAACAAGTGGGTTTGTTGACTG |
| 259 | gma-miR4410 | TATGTTGATCCGTATGAGTCGTAC |
| 260 | gma-miR169d | TGAGCCAAGGATGACTTGCCGGT |
| 261 | gma-miR1520q | ATTGACCAATCAGAACATGACACA |
| 262 | gma-miR172f | AGAATCTTGATGATGCTGCA |
| 263 | gma-miR171c | AGATATTGGTGCGGTTCAATC |
| 264 | gma-miR169e | AGCCAAGGATGACTTGCCGG |
| 265 | gma-miR394b | AGGTGGGCATACTGTCAACT |
| 266 | gma-miR4412-5p | TGTTGCGGGTATCTTTGCCTC |
| 267 | gma-miR4412-3p | AGTGGCGTAGATCCCCACAAC |
| 268 | gma-miR4413 | AAGAGAATTGTAAGTCACTG |
| 269 | gma-miR156g | ACAGAAGATAGAGAGCACAG |
| 270 | gma-miR159d | AGCTGCTTAGCTATGGATCCC |
| 271 | gma-miR4414 | AGCTGCTGACTCGTTGGCTC |
| 272 | gma-miR4416 | ACGGGTCGCTCTCACCTAGG |
| 273 | gma-miR396e | TTCCACAGCTTTCTTGAACTGT |
| 274 | gma-miR160b | TGCCTGGCTCCCTGTATGCC |
| 275 | gma-miR164b | TGGAGAAGCAGGGCACGTGC |
| 276 | gma-miR166h-5p | GGAATGTTGTTTGGCTCGAGG |
| 277 | gma-miR168b | TCGCTTGGTGCAGGTCGGG |
| 278 | gma-miR171d | TGATTGAGTCGTGTCAATATC |
| 279 | gma-miR319f | TTGGACTGAAGGGGCCTCTT |
| 280 | gma-miR408 | TGCACTGCCTCTTCCCTGGC |
| 281 | gma-miR2118a | TTGCCGATTCCACCCATTCCT |
| 282 | gma-miR482c-5p | ATTTGTGGGAATGGGCTGATTGG |
| 283 | gma-miR482c-3p | TTCCCAATTCCGCCCATTCCT |
| 284 | gma-miR530 | TGCATTTGCACCTGCACTTT |
| 285 | gma-miR862a | TGCTGGATGTCTTTGAAGGAAT |
| 286 | gma-miR1507c* | GAGGTGTTTGGGATGAGAGAA |
| 287 | gma-miR1507c | CCTCATTCCAAACATCATCT |
| 288 | gma-miR4992 | ATTCTAAGATGGTTTTTGTTAG |
| 289 | gma-miR4993 | GAGCGGCGGCGGTGGAGGATG |
| 290 | gma-miR4994 | GGTTAGCTCAAGGATCTCAC |
| 291 | gma-miR4995 | AGGCAGTGGCTTGGTTAAGGG |
| 292 | gma-miR4996 | TAGAAGCTCCCCATGTTCTC |
| 293 | gma-miR4997 | GATCGTCAAGCGCGAAGATGAGG |
| 294 | gma-miR4998 | AGTTTCGTGACTACAACTTCTG |
| 295 | gma-miR5030 | AGAACAATTTGTGTTTTACCGG |
| 296 | gma-miR5031 | TTAATGATTAACATCTAATTT |
| 297 | gma-miR1523b | TCATCGCTCCTGAGCTCACA |
| 298 | gma-miR5032 | AGAGCCACTTTTGGGTTCCCTAT |
| 299 | gma-miR5033 | GGCTGTACAAAAGGAAACTAC |
| 300 | gma-miR171h | ATTGAGACGAGCCGAATCAAT |
| 301 | gma-miR5034 | GGTACCCTTTCAGATAGTCTCA |
| 302 | gma-miR171i-5p | ATAAGAAAGCAATGCTCAAA |
| 303 | gma-miR171i-3p | TTGAGCCGTGCCAATATCACG |
| 304 | gma-miR5035 | CTTCTAAACATTTTTTCCCTTA |
| 305 | gma-miR5036 | AGAGGCCCTTGGGGAGGAGTAA |
| 306 | gma-miR5037 | GCCTCAAAGGCTTCCACTACTG |
| 307 | gma-miR1516b | AGCTTCTCTACAGAAAATATA |
| 308 | gma-miR169h | GGCGAGACATCTTGGCTCATT |
| 309 | gma-miR5038a | TGAGAATTTGGCCTCTGTCCA |
| 310 | gma-miR167h | ATCATGCTGGCAGCTTCAACTGGT |
| 311 | gma-miR1521b | GACTGTCACGTGTCATAATCATA |
| 312 | gma-miR5039 | CCCTTTTTTAATCGTTGCATG |
| 313 | gma-miR5040 | ATGATATATAACAAGCATGAG |
| 314 | gma-miR169i | CCGGTGCCATCCCGTCTCATA |
| 315 | gma-miR396f | AGCTTTCTTGAACTTCTTATGCCTA |
| 316 | gma-miR5041 | TTTCATCTTCAACTTGCTCAA |
| 317 | gma-miR396g | TTCTTGAACTTCTTATGCATC |
| 318 | gma-miR5042 | TATCTTGGATCACAGCCCCATT |
| 319 | gma-miR4372b | TAATAAAATCGTGACATGTAAC |
| 320 | gma-miR5043 | TGTCCCCTTCTCTGCACCACC |
| 321 | gma-miR5044 | GTAGTGGATGCCTAGAGGTCCA |
| 322 | gma-miR167i | TCATGCTGGCAGCTTCAACTGGT |
| 323 | gma-miR5368 | GGACAGTCTCAGGTAGACA |
| 324 | gma-miR5369 | TGAGAAAAGGAGGATGTCA |
| 325 | gma-miR862b | GCTGGATGTCTTTGAAGGA |
| 326 | gma-miR5037b | AACCCTCAAAGGCTTCCTAG |
| 327 | gma-miR5037c | AGTGGAACTTTGAGGCCTGC |
| 328 | gma-miR5370 | CTAAAGATTGTCCAAAAGGAA |
| 329 | gma-miR5371-5p | TAGGAATTAGTCACTCAGATC |
| 330 | gma-miR5371-3p | TCTCAGTGACTAATTTCTAGA |
| 331 | gma-miR5372 | TTGTTCGATAAAACTGTTGTG |
| 332 | gma-miR5373 | TCTCTTGATTCTAGATGATGT |
| 333 | gma-miR5374 | TTATAGTCTGACATCTGGAAT |
| 334 | gma-miR5375 | ACTATAGAAGTACTTGTGGAGC |
| 335 | gma-miR5376 | TGAAGATTTGAAGAATTTGGGA |
| 336 | gma-miR5377 | CTGAAGGATCGATGTAGAATGCT |
| 337 | gma-miR5378 | CATCTGAAGGATAGAACACATA |
| 338 | gma-miR5379 | ATGAAAATCATTCATTATGATATC |
| 339 | gma-miR5380a | GAAAATGAATGATGAGGATGGGGA |
| 340 | gma-miR171j | TATTGGCCTGGTTCACTCAGA |
| 341 | gma-miR408b-5p | CTGGGAACAGGCAGGGCACG |
| 342 | gma-miR3522 | AGACCAAATGAGCAGCTGA |
| 343 | gma-miR156k | TTGACAGAAGAGAGTGAGCAC |
| 344 | gma-miR159e-5p | GAGCTCCTTGAAGTCCAATT |
| 345 | gma-miR166i-5p | GGAATGTCGTCTGGTTCGAG |
| 346 | gma-miR166j-3p | TCGGACCAGGCTTCATTCCCG |
| 347 | gma-miR169k | CAGCCAAGAATGACTTGCCGG |
| 348 | gma-miR172g | GCAGCACCATCAAGATTCAC |
| 349 | gma-miR319g | TTGGACTGAAGGGAGCTCCTTC |
| 350 | gma-miR319i | TTGGACTGAAGGGGAGCTCCTTC |
| 351 | gma-miR396h | TCCACAGCTTTCTTGAACTG |
| 352 | gma-miR396i-3p | GTTCAATAAAGCTGTGGGAAG |
| 353 | gma-miR1512b | TAACTGGAAATTCTTAAAGCAT |
| 354 | gma-miR1513c | TATGAGAGAAAGCCATGAC |
| 355 | gma-miR4413b | TAAGAGAATTGTAAGTCACT |
| 356 | gma-miR5667 | AAACAGATCTAAATGGATTCC |
| 357 | gma-miR5669 | CAATGTAGTGTGGTAAGTGGTC |
| 358 | gma-miR5670 | CATCATACCATATTTGCTTCAT |
| 359 | gma-miR5672 | CATGGTAGTGGAAGAAATGGA |
| 360 | gma-miR5037d | CGGGAGCCTATGAAGGTTAAC |
| 361 | gma-miR5673 | CGTGGAATCTCGCGGAAGACAT |
| 362 | gma-miR1512c | TAACTGAACATTCTTAGAGCAT |
| 363 | gma-miR5675 | TAGAGACGACAACAATGGAAA |
| 364 | gma-miR4401b | TCAAAGACGTTGCTGAGGTAA |
| 365 | gma-miR5676 | TCGACACCATATGTAGAGGCAG |
| 366 | gma-miR5678 | TTCCATGATAAGATCTTTGAC |
| 367 | gma-miR5679 | TTGGTGACCCAGAAGAAGTTGA |
| 368 | gso-miR482a | TCTTCCCTACACCTCCCATAC |
| 369 | gso-miR3522b | TGAGACCAAATGAGCAGCTGAC |
| 370 | lja-miR2111* | GTCCTTAGGATGCAGATTACC |
| 371 | mtr-miR399b | TGCCAAAGGAGAGCTGCCCTG |
| 372 | mtr-miR399d | TGCCAAAGGAGAGCTGCCCTA |
| 373 | mtr-miR395a | ATGAAGTGTTTGGGGGAACTC |
| 374 | mtr-miR395b | ATGAAGTATTTGGGGGAACTC |
| 375 | mtr-miR399c | TGCCAAAGGAGATTTGCCCTG |
| 376 | mtr-miR399a | TGCCAAAGGAGATTTGCCCAG |
| 377 | mtr-miR171 | TGATTGAGTCGTGCCAATATC |
| 378 | mtr-miR395g | TTGAAGTGTTTGGGGGAACTC |
| 379 | mtr-miR395h | ATGAAGTGTTTGGGGGAACTT |
| 380 | mtr-miR395p | TTGAAGCGTTTGGGGGAACTC |
| 381 | mtr-miR166b | TCGGACCAGGCTTCATTCCTA |
| 382 | mtr-miR169d | AAGCCAAGGATGACTTGCCGG |
| 383 | mtr-miR169e | GGAGCCAAGGATGACTTGCCG |
| 384 | mtr-miR171b | TGATTGAGCCGCGTCAATATC |
| 385 | mtr-miR166c | TCGGACCAGGCTTCATTCCTC |
| 386 | mtr-miR166d | TCGGGCCAGGCTTCATCCCCC |
| 387 | mtr-miR169f | AAGCCAAGGATGACTTGCCTA |
| 388 | mtr-miR399j | CGCCAAAGAAGATTTGCCCCG |
| 389 | mtr-miR399k | TGCCAAAGAAGATTTGCCCTG |
| 390 | mtr-miR171c | TGATTGAGCCGTGCCAATATT |
| 391 | mtr-miR172 | AGAATCCTGATGATGCTGCAG |
| 392 | mtr-miR168 | TTGCTTGGTGCTGGTCGGGAA |
| 393 | mtr-miR156g | TTGACAGAAGATAGAGGGCAC |
| 394 | mtr-miR164d | TGGAGAAGCAGGGCACATGCT |
| 395 | mtr-miR171e | AGATTGAGCCGCGCCAATATC |
| 396 | mtr-miR169h | GAGCCAAAGATGACTTGCCGG |
| 397 | mtr-miR169i | TGAGCCAAAGATGACTTGCCG |
| 398 | mtr-miR169m | GAGCCAAGGATGACTTGCCGG |
| 399 | mtr-miR2086* | CCAGTTCTGCGTTCATGTCCC |
| 400 | mtr-miR1510b* | CCATGGATCCCTACCATGTGG |
| 401 | mtr-miR1510b | ACATGGTCGGTATCCCTGGAA |
| 402 | mtr-miR1507* | AGAGTTGTATGGAACGAAAGAT |
| 403 | mtr-miR1507 | CCTCGTTCCATACATCATCTAG |
| 404 | mtr-miR1510a* | TTGTCTTACCCATTCCTCCCA |
| 405 | mtr-miR1510a | CGGAGGATTAGGTAAAACAAC |
| 406 | mtr-miR1509 | TTAATCTAGGAAAATACGGTG |
| 407 | mtr-miR1509* | ACCGGATTTCCTTGATTAAAG |
| 408 | mtr-miR2087 | GAAGTAAAGAACCGGCTGCAG |
| 409 | mtr-miR2087* | CTGCAGTCGGTTTCTTACTTC |
| 410 | mtr-miR2088a | AGGCCTAGATTACATTGGAC |
| 411 | mtr-miR2088a* | TCCAATGTAATCTAGGTCTA |
| 412 | mtr-miR2089 | TTACCTATTCCACCAATTCCAT |
| 413 | mtr-miR2089* | AGGATTGGTGTAATAGGTAAAA |
| 414 | mtr-miR2119 | TCAAAGGGAGGTGTGGAGTAG |
| 415 | mtr-miR2118 | TTACCGATTCCACCCATTCCTA |
| 416 | mtr-miR2199 | TGATACACTAGCACGGATCAC |
| 417 | mtr-miR2585a | CAGGATTAGCGATTACAGGGAC |
| 418 | mtr-miR2586 | CGAGGAGTGTCCGTGCTTCAT |
| 419 | mtr-miR2587a | TTGACCGTTCATATGAACCCTG |
| 420 | mtr-miR2588a | TAACACTGTGCAACTAAGTCC |
| 421 | mtr-miR2589 | GGCATCCACGTGTGCTTCACCG |
| 422 | mtr-miR2590a | ATCTAAAGGTGATTATTGTGCC |
| 423 | mtr-miR2591 | GGAACTTCTACGGTACACCTGC |
| 424 | mtr-miR2592b | AAATGCTTGAGTCCTGTTGTT |
| 425 | mtr-miR2592d* | CAACAGGACTCAAGCATTTCGC |
| 426 | mtr-miR2592s | AAATGCTTGAGTCATGTTGTT |
| 427 | mtr-miR2593a | TTAAATGAATGAACCTAGAAT |
| 428 | mtr-miR2594a | CCATGGCCAAGGATGCCAGAG |
| 429 | mtr-miR2595 | TACATTTTCTTCTTTATGTCT |
| 430 | mtr-miR2596 | TCTATTTCATTGTTCCACACA |
| 431 | mtr-miR2597 | TTTGGTACTTCGTCGATTTGA |
| 432 | mtr-miR2111g | AGCCTCGGAGTGCGGATTATC |
| 433 | mtr-miR2598 | CTAAGGGTGATTATTCTGCCA |
| 434 | mtr-miR2599 | TGGGTACAAGGAATCTACTTT |
| 435 | mtr-miR2600 | ACATTAGCCAATCACAATGCC |
| 436 | mtr-miR2601 | TATTTGGTATCGCTTTGGTCCC |
| 437 | mtr-miR2602a | TGGCAGTGATTGCCACGTCAT |
| 438 | mtr-miR2603 | TTTGGTATTGGTCCCTGCACTT |
| 439 | mtr-miR2604 | TAATTTTTATGTGGGAGTGTT |
| 440 | mtr-miR2605 | ACTTAGTTTATATGACCTAC |
| 441 | mtr-miR2606a | TACAATTCCTTAGGTGCTTTT |
| 442 | mtr-miR2607 | ATGTGATTATGTGATAAGTGT |
| 443 | mtr-miR2608 | GTTGTACATATATCACTACTCT |
| 444 | mtr-miR2609a | TGGAAGTAATAGGTTCTCACT |
| 445 | mtr-miR2610a | AGATTGAGACTTGTATGGCTT |
| 446 | mtr-miR2611 | TATTTGTCAGTGTTTGATGAA |
| 447 | mtr-miR169q | TGAGCCAGGATGACTTGCCGG |
| 448 | mtr-miR2612a | TGATAGTGTCAACTAGTACAG |
| 449 | mtr-miR2613 | CGGTCGCCGGTGGTCAATGGT |
| 450 | mtr-miR2614 | CGGTTCGACTCGTTAGGTTC |
| 451 | mtr-miR2615a | CCTGATCGCATTTTAAAAGGC |
| 452 | mtr-miR2616 | ATTGGGTTTGGTTCGGGCGGAT |
| 453 | mtr-miR1509b | TTAATCTAGGAAATTACACTCG |
| 454 | mtr-miR2617a | TGTAGTGTAGCATGCCCGTT |
| 455 | mtr-miR2618a | GTGAATTCAGTTTACGTACGTT |
| 456 | mtr-miR2619 | ACATAGGAGGCTGTTTTGTAT |
| 457 | mtr-miR2620 | TTCTGATAGACACCGGCTCTGC |
| 458 | mtr-miR2621 | AGCTTGGGCTAGGAATTTGTGC |
| 459 | mtr-miR2622 | TTTGTGTGCCATCGTGAACTTA |
| 460 | mtr-miR2623 | TCGGCTGTACTGTCCTTCATG |
| 461 | mtr-miR2624a | CGAAAGACGAGGTTGCCGGCT |
| 462 | mtr-miR2625 | CCATCGTGCCACGTTACGATCC |
| 463 | mtr-miR2626 | AACGTCGGGATTTAGGGTGTT |
| 464 | mtr-miR2627 | TTTCGGTAGTTAACTGCTGAGG |
| 465 | mtr-miR2592a* | CCCGGCATTCATGTTTTCCT |
| 466 | mtr-miR2592a | GAAAAACATGAATGTCGAGCG |
| 467 | mtr-miR2628 | CATGAAAGAATGATGAGTAA |
| 468 | mtr-miR2629a | AGTTTTCCTCGGTAGTTAACT |
| 469 | mtr-miR2630a | TGGTTTTGGTCCTTGGTATTT |
| 470 | mtr-miR2631 | TGACACGCCACGTGGCACACT |
| 471 | mtr-miR2632a | CCTGAAGTTACTAATCCTTCCA |
| 472 | mtr-miR2633 | TGACATTTTGCTCCAGATTCA |
| 473 | mtr-miR2634 | TTTATTCTCAGTTTGTTGCTC |
| 474 | mtr-miR2635 | ATTATTGTCAACGTGACTAG |
| 475 | mtr-miR2636 | TTTGGTTAGTGTGCTGAATAT |
| 476 | mtr-miR2637 | AAATACTTCCTCTGATCACTG |
| 477 | mtr-miR2638a | ATGATTAATATTTGCAGTGGC |
| 478 | mtr-miR2639 | TAGTCGGCTTACGTCACCTTG |
| 479 | mtr-miR2640a | TTCCTTGCCGGAGCTGGACTAC |
| 480 | mtr-miR2641 | GTTTGATCCTTTACGTTTAT |
| 481 | mtr-miR2642 | ATGAGTTTCATCAAATCATGT |
| 482 | mtr-miR2643 | TTTGGGATCAGAAATTAGAGA |
| 483 | mtr-miR2644a | CACTTCAGATTGATGGTGTGT |
| 484 | mtr-miR2645 | TTTCTAGAGATGAGCATATAT |
| 485 | mtr-miR2646a | CATGACATTTAGTGATGATGT |
| 486 | mtr-miR2647a | ATTCACGGGGACGAACCTCCT |
| 487 | mtr-miR2648 | TAGCCAATGGGAATAACAGAT |
| 488 | mtr-miR2649 | AAAGGTGCCAATTATGAGTGT |
| 489 | mtr-miR2650 | AACTTAAATATGTTTTCAGTCC |
| 490 | mtr-miR2651 | TTTGATTGGTATGCCTGCATT |
| 491 | mtr-miR2652a | TATGCAGGGTGCATAAGGATT |
| 492 | mtr-miR2653a | TCACGCTGCTGTGAACATGAT |
| 493 | mtr-miR2654 | ATTCAGGGACAAAGTGTGCG |
| 494 | mtr-miR2655a | CGTTTAGGTCCCTTAACTTTA |
| 495 | mtr-miR2656a | AAGTTGCATAATCGAGTTGG |
| 496 | mtr-miR2657a | TGTTATTTCATCGATTTTGTTG |
| 497 | mtr-miR2658 | ATGTGACCTTGTATATGATC |
| 498 | mtr-miR2659a | CCATGGGTGCGACTTGGTAAG |
| 499 | mtr-miR2660a | TAAGACATCAGCTATAAGCTA |
| 500 | mtr-miR2661 | TAGGTTTGAGAAAATGGGCAG |
| 501 | mtr-miR2662 | GAGTAAAAATGTGAACCGAAT |
| 502 | mtr-miR2663 | TTAGAGAGGGCGTTACAATT |
| 503 | mtr-miR2664 | AATTGTGGTGGGTTGACAGTC |
| 504 | mtr-miR2665 | TGATTTCAGGTCAAGAATTGA |
| 505 | mtr-miR2666 | CGAAAGTGAGGATATCAAGGA |
| 506 | mtr-miR2667 | TCCTTGATCTGACGGCTACC |
| 507 | mtr-miR2668 | TTCATCCTTGCAATTAGGGGTC |
| 508 | mtr-miR2669 | AAAGTTCAGTCTTCATAGTATC |
| 509 | mtr-miR399q | TGCCAAAGGAGAGCTGCTCTT |
| 510 | mtr-miR2670a | CAAGAAGGTTGCTCACTATTT |
| 511 | mtr-miR2671j | TTAAAAGTTTCGTTTCGGTCC |
| 512 | mtr-miR2672 | TTAATCGACCAAGTGGGTACTA |
| 513 | mtr-miR2673a | CCTCTTCCTCTTCCTCTTCCAC |
| 514 | mtr-miR2674 | CACTCGCTTTGGAAGTCATGG |
| 515 | mtr-miR2675a | CGAGGCATATTTGCAGGGATT |
| 516 | mtr-miR2676a | CATTGTTTGGATAATAATTTG |
| 517 | mtr-miR2088b | TCCAATGTAATCTAGGTCTAC |
| 518 | mtr-miR2677 | TTTATTGATATTGCTAATAGAT |
| 519 | mtr-miR2678 | TGAAATTGTTGCGAGTGTCTT |
| 520 | mtr-miR2679a | CTTTTCACTTTCGAACGGGTG |
| 521 | mtr-miR2680a | TCCTCGGTACCTATGTTGAT |
| 522 | mtr-miR5204 | GCTGGAAGGTTTTGTAGGAAC |
| 523 | mtr-miR5205a | CATACAATTTGGGACGGAGGGAG |
| 524 | mtr-miR5205b | CTTATAATTAGGGACGGAGGGAGT |
| 525 | mtr-miR5205c | CTTATAATTAGGGACGGAGGTAGT |
| 526 | mtr-miR5206 | ATGGGATCCTGTTGGTGGGTTAC |
| 527 | mtr-miR5207 | CATTAATGTGGGTTTGGACGGTT |
| 528 | mtr-miR5208a | AACATGGATGTTGTGAGTTTGTT |
| 529 | mtr-miR5209 | CGAGGAGGCGGTATTGTTTGAA |
| 530 | mtr-miR5210 | TAAATGTGTTGGAATTAAGGTT |
| 531 | mtr-miR5211 | TCGCAGGAGTGATGGGACCGGC |
| 532 | mtr-miR5212 | TGGATTTCGTATTTCTTTGGTA |
| 533 | mtr-miR5213 | TACGTGTGTCTTCACCTCTGAA |
| 534 | mtr-miR5213* | CAGAGTGCAGATACACGCATC |
| 535 | mtr-miR5214 | TGATAGAGCTAGACCATCGGAG |
| 536 | mtr-miR5215 | AGGAGGATGAGCTACCTGCTT |
| 537 | mtr-miR2592ae | CAACAGGACTCAAGCATTTCG |
| 538 | mtr-miR2586b | CGGTGTCGTATCGGTGTTGGAC |
| 539 | mtr-miR5216a | TTAGGAGTGAAAAACGGTGGAA |
| 540 | mtr-miR5217 | AGGTCATTTTGAACGGTCGGAT |
| 541 | mtr-miR5218 | TGAGACTTGGTAGTAAGATGAT |
| 542 | mtr-miR5219a | TCATGGAATCTCAGCTGCTGCA |
| 543 | mtr-miR5221 | AGGAGAGATGGTGTTTTGACTT |
| 544 | mtr-miR5222 | TTACAGGAGAAGAATGTATGGC |
| 545 | mtr-miR5223a | CGTGGAATTTACTTGAAGATGC |
| 546 | mtr-miR5224a | TCGAGGACATGAGGGACGTTAT |
| 547 | mtr-miR5225 | TCAGTCGCAGGAGAGATGACAC |
| 548 | mtr-miR5226 | TTTGTACAACTTGGAGGATTCA |
| 549 | mtr-miR5224b | CGGAAGAGGATTGTCGAGGACA |
| 550 | mtr-miR5227 | TGAAGAGAAGAAGATTGATGAA |
| 551 | mtr-miR2592ap | AGGCTGGTTTAGATGAAGGTA |
| 552 | mtr-miR4414a | AGCTGCTGACTCGTTGGTTCA |
| 553 | mtr-miR4414a* | ATCCAACGATGCGGGAGCTGC |
| 554 | mtr-miR2670e | TCTCAACAGGACGGATCACTA |
| 555 | mtr-miR2670f | AGTGGTCTGTTAGGTTGGGGA |
| 556 | mtr-miR4414b | TGTGAATGATGCGGGAGCTAA |
| 557 | mtr-miR5228 | TCTGGTGTACAACTTGATGGA |
| 558 | mtr-miR5229a | TTAGCAGGAAGAGTGACTATG |
| 559 | mtr-miR5230 | CAAATCTTGAATCGATTGGCA |
| 560 | mtr-miR5231 | TTATGCAAGTAGATAAGCTCA |
| 561 | mtr-miR5232 | TACATGTCGCTCTCACCTGAA |
| 562 | mtr-miR5233 | GAGGAGGATGGCCGTCTGGAC |
| 563 | mtr-miR5234 | TTTTGTTGTGGATGGCAGAAG |
| 564 | mtr-miR5235a | ATAAGGTCAATGATTGGCGTG |
| 565 | mtr-miR5236a | TGAATTTCGGGCAGATTTGGT |
| 566 | mtr-miR5237 | TTCAAAAGATTTAGTTGGGAT |
| 567 | mtr-miR5238 | TGTAGAAAAAACAAAGGGCAA |
| 568 | mtr-miR2592bi | TGGAACATTGGGAATGCCGGT |
| 569 | mtr-miR482 | GGCATGGGATAGTAGGGAAGA |
| 570 | mtr-miR5239 | TGGGAGAAAAGATAGAATGTG |
| 571 | mtr-miR5240 | TTGAAAAAATTGTGGATTTGA |
| 572 | mtr-miR5241a | TGACTGAATGGAAGAGTGCAT |
| 573 | mtr-miR5242 | TTGTAGAAACAAGCGATGTCA |
| 574 | mtr-miR5243 | TGGGCAGAGAAATCGTGAGGC |
| 575 | mtr-miR5244 | TATCTCATGAAGATTGTTGGT |
| 576 | mtr-miR5245 | CATCGTAGAACACAGGCAGTA |
| 577 | mtr-miR5246 | TTGCAGACAGCTTTGAAGGTT |
| 578 | mtr-miR5247 | GCAGGAGCAAGCATCTGATGA |
| 579 | mtr-miR5248 | TTTTTAGTTGGCATGCATTCA |
| 580 | mtr-miR5249 | ACTTAGGGGGCAGTTTTGTAG |
| 581 | mtr-miR5250 | TGAGAATGTTAGATACGGAAC |
| 582 | mtr-miR5251 | AGTAGATCTAGTTGGTGTCTT |
| 583 | mtr-miR5252 | TGAGAGCTCACTGAAGTCTGC |
| 584 | mtr-miR2643b* | TCTAATCTCTGTTCCCAATTA |
| 585 | mtr-miR5253 | GATGAAAATGATTATGTTGGA |
| 586 | mtr-miR5254 | AGGAGGTGGAAGCATTTGTGA |
| 587 | mtr-miR5255 | TGACTTGATAGAGGACATGGG |
| 588 | mtr-miR5256 | TAATGGATTATGTAAGATTAA |
| 589 | mtr-miR2111t | ATCCTTGGAATGCAGATTATC |
| 590 | mtr-miR5257 | ACAAGTAGAACCTTTTTTCTG |
| 591 | mtr-miR5258 | TCAAGTGACAAGGAAGATCTT |
| 592 | mtr-miR5259 | CAAGGGGTATTGCGGAGGATA |
| 593 | mtr-miR5260 | TTTGTATTGTTGACATGGCTT |
| 594 | mtr-miR5261 | TCATTGTAGATGGCTTTGGCT |
| 595 | mtr-miR5262 | TCTGTCAGTAGACTCAATTTC |
| 596 | mtr-miR5263 | TGACTAAAACTAGTAACGGGG |
| 597 | mtr-miR172c* | GTAGCATCATCAAGATTCACA |
| 598 | mtr-miR160f | GCGTGAAGGGAGTCAAGCAGG |
| 599 | mtr-miR399r | TGCCAAAGAAGATTTGCCCCG |
| 600 | mtr-miR156j | TGACAGAAGAGGGTGAGCAC |
| 601 | mtr-miR5264 | TTGATCAAGGACTTTGCATC |
| 602 | mtr-miR5265 | AAGTGATGTTGGAATGGTTA |
| 603 | mtr-miR5266 | CTGGGGGACTGTCTGGGGCG |
| 604 | mtr-miR5267a | AGGCATTTGCTAGAATACACCCAC |
| 605 | mtr-miR5267l | AGGCATTTGCTAGGATACACCCAC |
| 606 | mtr-miR5268a | CCAGAGTGGAATGAAGATATGGTT |
| 607 | mtr-miR5269a | AAAGTGGTGGAACATACATTGATT |
| 608 | mtr-miR5269b | AAAGTGGTGGGACATACATTGATT |
| 609 | mtr-miR5270a | GAGGAGGAGTAGTTTTAGGTCATT |
| 610 | mtr-miR5271a | CGGATAATTGTGGTTACTAACGGT |
| 611 | mtr-miR5272a | GAATTGATTTATGTTTGGATACAC |
| 612 | mtr-miR5272f | GAATTGATTATGTTTGGATACACT |
| 613 | mtr-miR5273 | TAGGGGCTGTAGTTTGAGAAGAGG |
| 614 | mtr-miR2590g | AAATGAGACTGAAATCTAAAGGTG |
| 615 | mtr-miR2629h | GCAGAAGATCCTCGGCAGTTAACT |
| 616 | mtr-miR5274 | CGTTCTACAATATGACGGAGTGTA |
| 617 | mtr-miR2606d | AGTTAAGAACCATACAAAAAACAC |
| 618 | mtr-miR5275 | AGCTGGAGTCACATGCTTGAATTT |
| 619 | mtr-miR5276 | AGGGGGAGCACCTTGCTGGGGCAT |
| 620 | mtr-miR5208d | CATATTAGTCATATTTGTAGGCAT |
| 621 | mtr-miR5277 | AGGTTGTTTCTTGAAGTGCAAGGC |
| 622 | mtr-miR5278 | GAAATTATCTGCAGGAAATGTGAA |
| 623 | mtr-miR5279 | CGGAACCACTCGGATGACTCGGTT |
| 624 | mtr-miR5280 | TAATTAGAAACGGGCCGTGATGGG |
| 625 | mtr-miR5281a | CTCTTGTAAATAGGATCGGAGGGA |
| 626 | mtr-miR5281b | TCTTATAAATAGGACCGGAGGGAG |
| 627 | mtr-miR5282 | GACGGAATTAGAGAGGGATTTCAT |
| 628 | mtr-miR5283 | CGTGCGTATCGGGATGTATCGGAA |
| 629 | mtr-miR2600b | AAGCATTGTGGCATTGTGATTGGT |
| 630 | mtr-miR2600e | AAGCATTGTGGCATTGTGATTGGC |
| 631 | mtr-miR2590h | AGAATGACATGGCAGAATAATCAC |
| 632 | mtr-miR5284a | GAGGGACCAAAAGTGGAAGAATCT |
| 633 | mtr-miR5284b | GAGGGATCAAAAGTGGAAGAATCT |
| 634 | mtr-miR5284h | GAGGGATCAAAAGTGGAGGAATCT |
| 635 | mtr-miR5285a | TGGGACTTTGGGTAGAATTAGGCG |
| 636 | mtr-miR5286a | CAGGACAAACTGGAGGCAAGGGAC |
| 637 | mtr-miR5287a | TGCTTATATTAGTGACCGGAGGAT |
| 638 | mtr-miR5287b | TGCTTATAATAGTGATCGGAGGGT |
| 639 | mtr-miR5288 | CAGCATTGAAGAACATAGGGATTA |
| 640 | mtr-miR5289a | CGAGGAAAACTGAAAACTTCGGCA |
| 641 | mtr-miR5286b | ACAAACTGGAGGCAAGGGACAGGA |
| 642 | mtr-miR5290 | AATTTGGAGAGAGATAGACACATA |
| 643 | mtr-miR5291a | GTTTGATGGATGGATTGGATGGAT |
| 644 | mtr-miR5292a | ATTCAGATGATAGCAACAAAGAGC |
| 645 | mtr-miR5292b | GATTCAGATGATAGCAACAAAGAG |
| 646 | mtr-miR5293 | GATGAAGAAGTGGAAGGAAGAAGA |
| 647 | mtr-miR5294a | GCTAAACGGAATGAGGGTAGTCAT |
| 648 | mtr-miR5295a | TCGGCTCTGGGAATGAAAAGAGGC |
| 649 | mtr-miR5296 | ATTTTGTGTGGGTGTAAGAGGTGT |
| 650 | mtr-miR5297 | ATCGGGAAGTATCGGATAATTATT |
| 651 | mtr-miR5298a | TGGATATGATATGAAGATGAAGAA |
| 652 | mtr-miR5298b | TGATGGAGATGATATGAAGATGAA |
| 653 | mtr-miR5298d | TGGAGATGATATGAAGATGAAAAA |
| 654 | mtr-miR5299 | TTCATTGGTATTGTAAAGCGACAT |
| 655 | mtr-miR5554a | TGTGCATCTTGAACAATGGTAT |
| 656 | mtr-miR5554a* | ACCATCGTTGCAGATGCTCATC |
| 657 | mtr-miR5555 | TAAGAGTATAATATGACTTTG |
| 658 | mtr-miR5555* | AAGTCGTATTACACTCTTAGA |
| 659 | mtr-miR2619b | ATATGTTTTGATTCTTTGGCA |
| 660 | mtr-miR2619b* | CCAAAGAATCAATACATAGGG |
| 661 | mtr-miR2592bl | TGGCAAGTTTGAATTTACCTCA |
| 662 | mtr-miR2592bl* | GAGTAATTCAAACTTGTTAAA |
| 663 | mtr-miR5274b | ATATGACGGAGTGTAAATGCC |
| 664 | mtr-miR5274b* | CATTTACACTCCGTCATATTG |
| 665 | mtr-miR5556* | TGGAATTCTTCCGCCATCCAA |
| 666 | mtr-miR5556 | TGATGACGGAAGAAATCCAAA |
| 667 | mtr-miR2592bm* | CTCGGCATTCATGTTTTTCCTT |
| 668 | mtr-miR2592bm | GGAAAACATGAATGTCGGGTG |
| 669 | mtr-miR5557* | AACAAGTACTAAGGAAGCACA |
| 670 | mtr-miR5557 | TGCTTCCTTAGTACTTGTTGA |
| 671 | mtr-miR5558 | TTTTCCAATTCTAAGTCTATC |
| 672 | mtr-miR5558* | TAGATTTAGAATTAGAAAAGC |
| 673 | mtr-miR5559 | TACTTGGTGAATTGTTGGATC |
| 674 | mtr-miR5559* | TCTAATTATTCACCAAGTAAA |
| 675 | mtr-miR5560* | CTCATTCACTCAGCCGGTACA |
| 676 | mtr-miR5560 | TGCCGGCTCAATGAATGCGGAG |
| 677 | mtr-miR5561 | CATTTGGAGAGACATAGACAA |
| 678 | mtr-miR5561* | GTCTATCTCTCTCTAAATGGA |
| 679 | mtr-miR5562 | TGTGGAGTCTTTTGCATGAAG |
| 680 | mtr-miR5562* | ATGTGGAGAAGGCTGCAAC |
| 681 | mtr-miR5563 | TGATATCAGGCAACTCGGTCC |
| 682 | mtr-miR5563* | ACTGAGTTGCCTAATGTCGTT |
| 683 | mtr-miR167b* | GATCATGTTGGAGCTTCACC |
| 684 | mtr-miR168c* | CCCGCCTTGCATCAACTGAAT |
| 685 | mtr-miR408* | ACAGGGAACATGCAGAGCATG |
| 686 | mtr-miR2111v* | AGCCTTGGAATGCAGATTATC |
| 687 | pvu-miR159a.2 | CTTCCATATCTGGGGAGCTTC |
| 688 | pvu-miR1514a | TTCATTTTGAAAATAGGCATTG |
| 689 | pvu-miR482* | GGAATGGGCTGATTGGGAAGCA |
| 690 | pvu-miR482 | TCTTCCCAATTCCGCCCATTCC |
| 691 | pvu-miR319c | TTGGACTGAAGGGAGCTCCTT |
| 692 | vun-miR319b | CTTGGACTGAAGGGAGCTCCT |
| 693 | vun-miR164 | TGGAGAAGGGGAGCACGTGCA |
| 694 | vun-miR399b | TGCCAAAGGAGAATTGCCCTG |

a. mtr / p mean “*Medicago truncatula*”; b. vun means “*Vigna unguiculata*”; c. aau means “*Acacia auriculiformis*”; d ahy means “*Arachis hypogaea*”; e. amg means “Acacia mangium”; f. gma / Gma mean “*Glycine max*”; g. gso means “*Glycine soja*”; h. lja means “*Lotus japonicus*”; i. pvu means “*Phaseolus vulgaris*”.
