# Supplementary material for: Identification and Comparative Analysis of Cadmium Tolerance-Associated miRNAs and Their Targets in Two Soybean Genotypes
Source: PLoS One. 2013 Dec 10;8(12):e81471. doi: 10.1371/journal.pone.0081471 (PMC3867309; doi:10.1371/journal.pone.0081471)
Supplement: Table S2 — The stem–loop RT and qRT-PCR primer sequences for miRNAs expression analysis. (DOC) [file pone.0081471.s004.doc]

**Table S2. The stem–loop RT and qRT-PCR primer sequences for miRNAs expression analysis.**

| name | primer | sequence (5’-3’) |
| --- | --- | --- |
| universal | reverse primer | CAGTGCAGGGTCCGAGGTAT |
| F-box | forward primer | ATGGTCGCCGTTTAGAACAC |
|  | reverse primer | GGGATAACCAGTGCAGAAGC |
| gma-miR1509b | stem-loop RT primer | GTCGTATCCAGTGCAGGGTCCGAGGTATTCGCACTGGATACGACAACCGT |
|  | forward primer | GCGGCGTTAATCAAGGAAATC |
| gma-miR3522 | stem-loop RT primer | GTCGTATCCAGTGCAGGGTCCGAGGTATTCGCACTGGATACGACTCAGCT |
|  | forward primer | AGCGGAGACCAAATGAGCA |
| gma-miR396a-3p | stem-loop RT primer | GTCGTATCCAGTGCAGGGTCCGAGGTATTCGCACTGGATACGACCTTCCC |
|  | forward primer | GCGCCGTTCAATAAAGCTGT |
| gma-miR396b-5p | stem-loop RT primer | GTCGTATCCAGTGCAGGGTCCGAGGTATTCGCACTGGATACGACAAGTTC |
|  | forward primer | CCGGTTCCACAGCTTTCTTG |
| gma-miR397a | stem-loop RT primer | GTCGTATCCAGTGCAGGGTCCGAGGTATTCGCACTGGATACGACCATCAA |
|  | forward primer | TTGAGTCATTGAGTGCAGCGA |
| gma-miR398c | stem-loop RT primer | GTCGTATCCAGTGCAGGGTCCGAGGTATTCGCACTGGATACGACCAGGGGC |
|  | forward primer | TGCGCATGTGTTCTCAGGTC |
| gma-miR408 | stem-loop RT primer | GTCGTATCCAGTGCAGGGTCCGAGGTATTCGCACTGGATACGACGCCAGG |
|  | forward primer | CTTGCTTGCACTGCCTCTTC |
| gma-miR408b-5p | stem-loop RT primer | GTCGTATCCAGTGCAGGGTCCGAGGTATTCGCACTGGATACGACCGTGCC |
|  | forward primer | GAGCTTCTGGGAACAGGCAG |
| gma-miR4996 | stem-loop RT primer | GTCGTATCCAGTGCAGGGTCCGAGGTATTCGCACTGGATACGACGAGAAC |
|  | forward primer | TCGCTAGAAGCTCCCCATGT |
| gma-miR5037b | stem-loop RT primer | GTCGTATCCAGTGCAGGGTCCGAGGTATTCGCACTGGATACGACCTAGGA |
|  | forward primer | CAGTGCAACCCTCAAAGGCT |
| PC-15-5p | stem-loop RT primer | GTCGTATCCAGTGCAGGGTCCGAGGTATTCGCACTGGATACGACTCCTAA |
|  | forward primer | GCGTCCGTTGTAGTCTAGTTGG |
| Gma-m040-5p | stem-loop RT primer | GTCGTATCCAGTGCAGGGTCCGAGGTATTCGCACTGGATACGACTGGTGC |
|  | forward primer | CGAACAAGCTCAGGAGGGATA |
| gma-miR1535b | stem-loop RT primer | GTCGTATCCAGTGCAGGGTCCGAGGTATTCGCACTGGATACGACCTAGAC |
|  | forward primer | CTGCGTCTTGTTTGTGGTGAT |
| gma-miR319c | stem-loop RT primer | GTCGTATCCAGTGCAGGGTCCGAGGTATTCGCACTGGATACGACAGGAGC |
|  | forward primer | GCGACGATTGGACTGAAAGG |
| gma-miR4403 | stem-loop RT primer | GTCGTATCCAGTGCAGGGTCCGAGGTATTCGCACTGGATACGACGTCCGT |
|  | forward primer | ACACGGACACCGAACACGA |
| Vun78330_1521_100 | stem-loop RT primer | GTCGTATCCAGTGCAGGGTCCGAGGTATTCGCACTGGATACGACAGGAGC |
|  | forward primer | AGGCGTTTGGACTGAAGGG |
