# Supplementary material for: Identification and Comparative Analysis of Cadmium Tolerance-Associated miRNAs and Their Targets in Two Soybean Genotypes
Source: PLoS One. 2013 Dec 10;8(12):e81471. doi: 10.1371/journal.pone.0081471 (PMC3867309; doi:10.1371/journal.pone.0081471)
Supplement: Table S3 — The primer sequences for qRT-PCR analysis of target genes. (DOC) [file pone.0081471.s005.doc]

**Table S3. The primer sequences for qRT-PCR analysis of target genes.**

| miRNA | Target gene | primer | sequence (5’-3’) |
| --- | --- | --- | --- |
|  | F-box | forward primer | ATGGTCGCCGTTTAGAACAC |
|  |  | reverse primer | GGGATAACCAGTGCAGAAGC |
| gma-miR397a | Glyma18g42520.1 | forward primer | CCAAAACTCTGGCAAATACAC |
|  |  | reverse primer | TGAAGTAATGAGCCTGAAGCA |
| gma-miR408 | Glyma03g26060.2 | forward primer | AGCAACAGCATCAAGAACTACC |
|  |  | reverse primer | CCTGAAATGGGGCACAAGA |
|  | Glyma08g13510.1 | forward primer | AGAGGAGCCAAAGTGTATCGG |
|  |  | reverse primer | CCAGACTCGCAGTGACCAAC |
| gma-miR398c | Glyma14g39910.1 | forward primer | AGATGCTGGGACTGAGACTAATG |
|  |  | reverse primer | GCTTGACAAATGGGAGGAGG |
|  | Glyma06g19680.1 | forward primer | ATCGTCTTTATTGTAGGCACCA |
|  |  | reverse primer | AGTCACCCCTTCATCATTTTC |
| gma-miR1509b | Glyma18g03980.2 | forward primer | ATGGTGGTCTGCTGGCTGTG |
|  |  | reverse primer | AGTGGGTCTCCTTATTCGCATTT |
| gma-miR396b-5p | Glyma15g19460.1 | forward primer | ATCCTCAACACTATGGCTCC |
|  |  | reverse primer | GCTCATCCACCTCCTCTTT |
|  | Glyma17g35090.1 | forward primer | TCACGTTGTCACCACTTAGGC |
|  |  | reverse primer | TCTCCAAGAGGACCACCCA |
| Vun78330_1521_100 | Glyma03g38120.1 | forward primer | CAATCAATAATGGTGGGAGG |
|  |  | reverse primer | CTGGCAAACTTTCAATGGC |
|  | Glyma19g40720.1 | forward primer | CCATTATGACTTGCTCCTTTTG |
|  |  | reverse primer | CTGTGGTATGACTCCATTTGC |
