# Supplementary material for: Identification and Comparative Analysis of Cadmium Tolerance-Associated miRNAs and Their Targets in Two Soybean Genotypes
Source: PLoS One. 2013 Dec 10;8(12):e81471. doi: 10.1371/journal.pone.0081471 (PMC3867309; doi:10.1371/journal.pone.0081471)
Supplement: Table S4 — The miRNAs from other species show strong signals (signal >1000) in soybean. (DOC) [file pone.0081471.s006.doc]

**Table S4. The miRNAs from other species show strong signals (signal > 1000**) in soybean.

| miRNA | Huaxia3 | | | | Zhonghuang24 | | | |  |
| --- | --- | --- | --- | --- | --- | --- | --- | --- | --- |
|  | CK | Cd | fold change log2(Cd/CK) | p-value | CK | Cd | fold change log2(Cd/CK) | p-value | mature Sequence (5' to 3') |
| PN-miR166_L-1 | 9,776 | 13,336 | 0.45 | 7.22E-03 | 9,139 | 10,507 | 0.20 | 2.56E-01 | CGGACCAGGCUUCAUUCCCC |
| PN-miR164a_L-1 | 4,057 | 4,890 | 0.27 | 8.68E-03 | 3,519 | 3,460 | -0.02 | 6.88E-01 | GGAGAAGCAGGGCACGUGCA |
| aau-miR319 | 8,600 | 6,569 | -0.39 | 3.38E-03 | 7,460 | 4,779 | -0.64 | 3.62E-02 | UUGGACUGAAGGGAGCUCCCU |
| PC-34-5p | 13,246 | 8,304 | -0.67 | 7.27E-03 | 8,180 | 6,408 | -0.35 | 1.08E-02 | GCUGGUUUGCCCGAGA |
| ahy-miR167-5p | 1,512 | 1,672 | 0.14 | 7.40E-02 | 1,235 | 1,900 | 0.62 | 4.84E-03 | UGAAGCUGCCAGCAUGAUCUU |
| gso-mir1510b-p5 | 676 | 743 | 0.14 | 1.37E-01 | 857 | 1,535 | 0.84 | 3.71E-03 | AGGGAUAGGUAAAACAACUAC |
| PN-miR160c_1ss15AC | 1,659 | 2,014 | 0.28 | 4.03E-02 | 1,562 | 1,947 | 0.32 | 5.94E-03 | UGCCUGGCUCCCUGCAUGCCA |
| gso-miR2109 | 1,395 | 1,348 | -0.05 | 5.93E-01 | 476 | 722 | 0.60 | 6.39E-04 | UGCGAGUGUCUUCGCCUCUGA |
| ahy-miR156a | 9,085 | 9,110 | 0.00 | 9.28E-01 | 10,927 | 7,751 | -0.50 | 4.58E-03 | UGACAGAAGAGAGAGAGCAC |
| ahy-miR156c | 8,886 | 8,972 | 0.01 | 7.80E-01 | 10,336 | 7,901 | -0.39 | 3.34E-03 | UUGACAGAAGAGAGAGAGCAC |
| aau-miR396 | 5,031 | 4,691 | -0.10 | 2.05E-01 | 11,612 | 6,272 | -0.89 | 1.35E-04 | UUCCACAGCUUUCUUGAACUG |
| PC-33-3p | 1,588 | 1,488 | -0.09 | 4.71E-01 | 1,801 | 1,182 | -0.61 | 5.78E-03 | GGAGAACAAAGAAGCAGCUAAAUUC |
| PN-mir156f-p3 | 988 | 879 | -0.17 | 1.02E-01 | 2,857 | 1,560 | -0.87 | 7.76E-04 | GCUCUCUCUUCCUCUGUCAUC |
| PN-miR5072_L-4 | 7,434 | 5,216 | -0.51 | 3.32E-02 | 5,856 | 3,354 | -0.80 | 7.55E-04 | UCCCCAGCGGAGUCGCCA |
