# Supplementary material for: Identification and Comparative Analysis of Cadmium Tolerance-Associated miRNAs and Their Targets in Two Soybean Genotypes
Source: PLoS One. 2013 Dec 10;8(12):e81471. doi: 10.1371/journal.pone.0081471 (PMC3867309; doi:10.1371/journal.pone.0081471)
Supplement: Table S6 — Expression profiles for ten target genes analysed in HX3-CK and ZH24-CK. (DOC) [file pone.0081471.s008.doc]

**Table S6. Expression profiles for ten target genes analysed in HX3-CK and ZH24-CK.**

| mRNA | HX3-CK | | ZH24-CK | |
| --- | --- | --- | --- | --- |
|  | Expression | SD | Expression | SD |
| Glyma18g42520.1 | 1 | 0.045335057 | 0.926500695 | 0.043766856 |
| Glyma03g26060.2 | 1 | 0.025344909 | 0.749567829 | 0.031534377 |
| Glyma08g13510.1 | 1 | 0.039557316 | 1.916277267 | 0.145211904 |
| Glyma14g39910.1 | 1 | 0.071464292 | 1.800685796 | 0.117527472 |
| Glyma06g19680.1 | 1 | 0.029530175 | 0.717987624 | 0.029119837 |
| Glyma18g03980.2 | 1 | 0.026793399 | 0.765464405 | 0.031235395 |
| Glyma15g19460.1 | 1 | 0.042997612 | 0.568992789 | 0.033040182 |
| Glyma17g35090.1 | 1 | 0.05738896 | 0.389413704 | 0.01773424 |
| Glyma03g38120.1 | 1 | 0.050941979 | 0.76141234 | 0.057555127 |
| Glyma19g40720.1 | 1 | 0.046194894 | 0.829154738 | 0.065801383 |
