# Supplementary material for: Identification and Comparative Analysis of Cadmium Tolerance-Associated miRNAs and Their Targets in Two Soybean Genotypes
Source: PLoS One. 2013 Dec 10;8(12):e81471. doi: 10.1371/journal.pone.0081471 (PMC3867309; doi:10.1371/journal.pone.0081471)
Supplement: Table S7 — Expression profiles for ten target genes analysed in HX3-Cd and ZH24-Cd. (DOC) [file pone.0081471.s009.doc]

**Table S7. Expression profiles for ten target genes analysed in HX3-Cd and ZH24-Cd.**

| mRNA | HX3-Cd | | ZH24-Cd | |
| --- | --- | --- | --- | --- |
|  | Expression | SD | Expression | SD |
| Glyma18g42520.1 | 1 | 0.044648337 | 1.629121168 | 0.069325156 |
| Glyma03g26060.2 | 1 | 0.013638966 | 0.869003346 | 0.055223981 |
| Glyma08g13510.1 | 1 | 0.062341941 | 1.926455033 | 0.114403194 |
| Glyma14g39910.1 | 1 | 0.03943287 | 3.253240437 | 0.177188127 |
| Glyma06g19680.1 | 1 | 0.015080357 | 0.723905326 | 0.061695708 |
| Glyma18g03980.2 | 1 | 0.028139806 | 0.852207398 | 0.045607157 |
| Glyma15g19460.1 | 1 | 0.034451236 | 0.417434081 | 0.038470306 |
| Glyma17g35090.1 | 1 | 0.025280069 | 0.291540138 | 0.016205163 |
| Glyma03g38120.1 | 1 | 0.029903567 | 0.957216239 | 0.062667764 |
| Glyma19g40720.1 | 1 | 0.030577218 | 0.870352252 | 0.065115933 |
